# Integrated multi-omics analysis reveals shared IFN-γ-associated monocyte signatures in primary Sjögren’s syndrome and Alzheimer’s disease

**DOI:** 10.3389/fimmu.2026.1923114

**Published:** 2026-09-09

**Authors:** Xiaohan Zhang, Shaofu Yan, Minghui Xu, Yanxin Zhang, Wenqi Tan, Tingjun Li, Jinyan Si, Ping Sun

**Affiliations:** 1 Hebei Medical University-Galway University Stem Cell Research Center, Hebei Medical University, Shijiazhuang, Hebei, China; 2 Shanxi Medical University School and Hospital of Stomatology, Taiyuan, China; 3 The First People’s Hospital of Jinzhong, Jinzhong, Shanxi, China; 4 The Fifth Clinical Medical College of Shanxi Medical University, Taiyuan, China; 5 Shanxi Oral Health Prevention and Control Technology Innovation Center, Taiyuan, China

**Keywords:** Alzheimer’s disease, epigenetic regulation, IFN-γ signaling, monocytes, OAS3, primary Sjögren’s syndrome

## Abstract

**Background:**

Primary Sjögren’s syndrome (pSS) is epidemiologically associated with an increased risk of Alzheimer’s disease (AD); however, the shared IFN-γ-related immune mechanisms underlying this association remain poorly understood.

**Methods:**

Peripheral blood transcriptomic, single-cell transcriptomic, and epigenomic datasets from patients with AD and pSS were integrated and analyzed. These findings were further validated using peripheral blood samples from patients and *in vitro* IFN-γ stimulation experiments to systematically characterize the shared IFN-γ-related immune features.

**Results:**

Integrated multi-omics analysis revealed shared IFN-γ-related immune signatures between pSS and AD, with transcriptional responses predominantly enriched in CD14^+^ and CD16^+^ monocytes. Among these, OAS3 was consistently upregulated in both monocyte subsets. Western blot analysis further confirmed elevated OAS3 protein expression in the peripheral blood of patients with pSS and AD. Integrated analysis of public multi-omics datasets together with *in vitro* IFN-γ stimulation experiments showed a time-associated increase in OAS3 expression following IFN-γ stimulation, which remained consistently induced even under low-dose stimulation. Although the IFN-γ-associated inflammatory transcriptional profiles differed between glucose and galactose culture conditions, OAS3 expression remained consistently induced under both metabolic conditions. ATAC-seq and CUT&Tag analyses showed that IFN-γ stimulation increased chromatin accessibility at the OAS3 promoter and enhanced H3K4me1 enrichment, a process that may be associated with activation of the JAK/STAT signaling pathway. Exploratory analysis of a publicly available OAS3-knockout THP-1 dataset revealed altered enrichment of immune- and neurodegeneration-related pathways.

**Conclusions:**

This study demonstrates that pSS and AD share IFN-γ-related immune-inflammatory features and identifies OAS3 as a candidate IFN-γ-responsive molecule that exhibits sustained responsiveness to IFN-γ stimulation. These findings provide new insights into the shared IFN-γ-associated immune features of pSS and AD and support the potential of OAS3 as a peripheral biomarker for future mechanistic and translational studies.

## 1 Introduction

Alzheimer’s disease (AD) is a chronic neurodegenerative disorder characterized by progressive cognitive decline and neuronal loss. With the rapid aging of the global population, its prevalence continues to increase, posing a major public health challenge worldwide (1–4). Although amyloid-β (Aβ) deposition, tau hyperphosphorylation, and neuroinflammation have been established as key pathological processes contributing to AD pathogenesis, the underlying mechanisms remain incompletely understood owing to the multifactorial nature of the disease and the lack of effective disease-modifying therapies (5). Increasing evidence has highlighted the critical role of peripheral immune dysregulation in AD. Persistent peripheral inflammation promotes immune cell activation and inflammatory cytokine release and is closely associated with Aβ deposition, tau pathology, and neuronal injury (6–9).

Primary Sjögren’s syndrome (pSS) is a prototypical systemic autoimmune disease characterized by chronic inflammation and lymphocytic infiltration of the exocrine glands, with frequent involvement of multiple organ systems (10). In addition to exocrine gland dysfunction, neurological manifestations are common in patients with pSS and are closely associated with disease activity (11–13). Epidemiological studies have further demonstrated that patients with pSS have a significantly increased risk of developing AD (14), suggesting that these two diseases may share common immunopathological mechanisms. However, studies investigating the mechanisms underlying their comorbidity remain limited, and the shared immune regulatory network observed in pSS and AD has yet to be fully elucidated (15).

Interferon (IFN) signaling is a central pathogenic pathway in pSS, encompassing both type I and type II interferon responses (16, 17). Among these, type II interferon (IFN-γ) induces the expression of interferon-stimulated genes (ISGs) primarily through the Janus kinase/signal transducer and activator of transcription (JAK/STAT) signaling pathway and regulates the inflammatory functions of innate immune cells, including monocytes and macrophages (18–20). In recent years, the role of IFN-γ in AD has also attracted considerable attention. IFN-γ has been shown to promote the activation of monocytes, macrophages, and microglia, enhance the expression of inflammation-associated genes, and contribute to neuroinflammatory processes (21, 22). Conversely, under specific conditions, IFN-γ may exert neuroprotective effects by modulating microglial metabolism and phagocytic activity (23). Nevertheless, whether IFN-γ contributes to the shared immune characteristics of pSS and AD, and the roles of its downstream effector molecules in this process, remain largely unexplored.

To address these questions, we performed an integrative multi-omics analysis of peripheral blood transcriptomic, single-cell RNA sequencing (scRNA-seq), and epigenomic datasets from patients with AD and pSS. These findings were further validated using peripheral blood samples from patients and *in vitro* IFN-γ stimulation experiments. Our study aimed to systematically characterize the shared IFN-γ-related immune features and their associated transcriptional and chromatin patterns, thereby providing new insights into the shared peripheral immune characteristics between pSS and AD, as well as the common immune signatures shared by autoimmune and neurodegenerative diseases.

## 2 Methods

### 2.1 Study design

**Figure 1:**
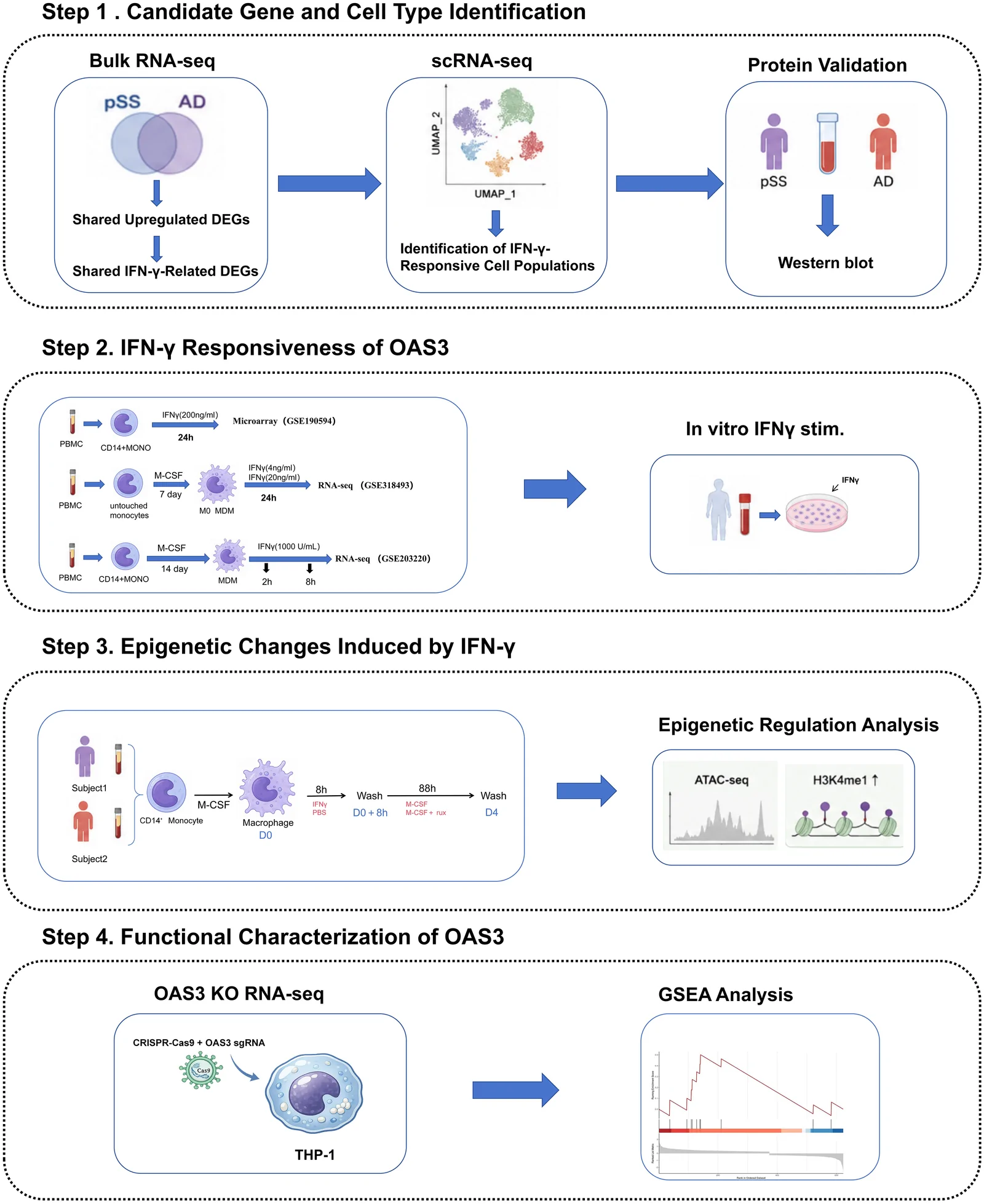
Study design and overall analytical workflow. The study comprised four sequential stages. Step 1: Identification of shared IFN-γ-related candidate genes and their predominant responsive cell types. Bulk RNA sequencing datasets of peripheral blood from patients with AD and pSS were integrated to identify shared IFN-γ-related DEGs. scRNA-seq data were subsequently analyzed to determine the predominant cell types expressing the candidate genes, and protein expression levels were further validated by Western blot analysis. Step 2: Characterization of the transcriptional response of OAS3 to IFN-γ stimulation. Publicly available transcriptomic datasets of IFN-γ stimulation, together with *in vitro* IFN-γ stimulation experiments, were integrated to characterize the expression dynamics of OAS3 under different stimulation durations, IFN-γ concentrations, and metabolic conditions. Step 3: Epigenetic characterization of IFN-γ-induced OAS3 regulation. ATAC-seq and H3K4me1 CUT&Tag datasets were analyzed to investigate dynamic changes in chromatin accessibility and H3K4me1 enrichment at the OAS3 locus following IFN-γ stimulation. Step 4: Transcriptomic analysis following OAS3 knockout. GSEA was performed using RNA-seq data generated from CRISPR/Cas9-mediated OAS3-knockout THP-1 cells to evaluate biological pathways altered upon OAS3 deficiency.

The overall study workflow consisted of four stages (**Figure 1**):

1. Identification of shared IFN-γ-related candidate genes and major responsive cell populations. Peripheral blood transcriptomic datasets from patients with AD and pSS were integrated to identify shared IFN-γ-related candidate genes. scRNA-seq data were subsequently analyzed to identify the major cell populations enriched for IFN-γ-related transcriptional responses. Candidate gene expression at the protein level was further validated by Western blotting.
2. Characterization of the transcriptional response of OAS3 to IFN-γ stimulation. Publicly available transcriptomic datasets of IFN-γ stimulation were integrated with *in vitro* stimulation experiments to evaluate OAS3 expression under different stimulation durations, IFN-γ concentrations, and metabolic conditions.
3. Analysis of IFN-γ-induced epigenetic alterations associated with OAS3. Publicly available assay for transposase-accessible chromatin using sequencing (ATAC-seq) and cleavage under targets and tagmentation (CUT&Tag) datasets were analyzed to investigate dynamic changes in chromatin accessibility and H3K4me1 enrichment at the OAS3 locus following IFN-γ stimulation.
4. Transcriptomic profiling following OAS3 knockout. RNA sequencing data from OAS3-knockout cells were subjected to Gene Set Enrichment Analysis (GSEA) to assess pathway alterations associated with OAS3 deficiency.

### 2.2 Data sources

IFN-γ-related genes were obtained from the Gene Ontology (GO) database (GO:0034341, response to interferon-gamma) and the HALLMARK_INTERFERON_GAMMA_RESPONSE gene set in the Molecular Signatures Database (MSigDB) (24, 25). After integrating the two gene lists and removing duplicates, a total of 315 IFN-γ-related genes were identified (**Supplementary Table 1**) (26). This unified 315-gene IFN-γ signature was used throughout the study for subsequent analyses, including: (1) identifying IFN-γ-related candidate genes by intersecting the shared upregulated differentially expressed genes (DEGs) between AD and pSS with the IFN-γ-related gene set; and (2) calculating the IFN-γ score for individual cells in single-cell transcriptomic datasets using the AddModuleScore algorithm to quantify IFN-γ-related transcriptional responses.

All other publicly available datasets were retrieved from the Gene Expression Omnibus (GEO) database (https://www.ncbi.nlm.nih.gov/geo/) (27). Datasets were selected according to the objectives of each analysis, and the corresponding expression matrices and platform annotation files were downloaded for subsequent analyses. Detailed information for each dataset, including GEO ID, data type, species, dataset description, number of cases, number of controls, and PMID, is summarized in **Supplementary Table 2**.

### 2.3 Transcriptomic analysis

Publicly available transcriptomic datasets were downloaded from the GEO database. Detailed information on all included datasets is provided in **Supplementary Table 2**. Different analytical strategies were applied according to the data type and format of each dataset.

For datasets providing raw RNA-seq count matrices (GSE248417, GSE203220, and GSE290458) (28–30), differential expression analysis was performed using the DESeq2 package in R. Raw counts were normalized using the DESeq2 size factor method, followed by differential expression analysis based on a negative binomial generalized linear model. P values were adjusted for multiple testing using the Benjamini–Hochberg (BH) procedure (31).

For datasets providing normalized expression matrices (GSE318493) (32, 33), differential expression analysis was performed using the limma package in R. The normalized FPKM expression matrix provided by the original authors was log_2_-transformed before analysis. Linear modeling with empirical Bayes moderation was applied, and P values were adjusted using the BH procedure (34).

For the OAS3-knockout dataset GSE113363, which comprised one WT sample and two independent OAS3-KO clones, limma was used only to estimate gene-level log_2_ fold changes for generation of the pre-ranked gene list used in the exploratory GSEA. Because of the limited biological replication, gene-level differential expression statistics from this dataset were not interpreted as formal statistical inference.

For the GSE294918 dataset (35), differential expression analysis was not performed because only a single biological sample was available. Instead, normalized expression values were used descriptively to illustrate OAS3 expression dynamics under different treatment conditions without statistical testing.

A false discovery rate (FDR) < 0.05 and an absolute log_2_ fold change (|log_2_FC|) ≥ 0.5 were used as the initial criteria for identifying DEGs for exploratory candidate gene screening. Candidate genes were subsequently prioritized through independent validation across multiple datasets and experimental analyses.

### 2.4 Microarray gene expression analysis

Publicly available microarray expression datasets GSE84844 and GSE190594 were downloaded from the GEO database (36, 37). Detailed information on these datasets is provided in **Supplementary Table 2**. As both studies provided author-processed expression matrices, the normalized expression data were used directly for downstream analyses without additional background correction or normalization.

Probe-to-gene annotation was performed according to the corresponding platform annotation files. Probes without gene annotations or those that could not be uniquely mapped to a single gene were excluded. For genes represented by multiple probes, the probe with the largest expression variance across samples was retained as the representative probe, thereby generating a gene-level expression matrix.

Differential expression analysis was performed using the limma package in R. Linear models combined with an empirical Bayes approach were applied for statistical inference (34). P values were adjusted for multiple testing using the BH procedure. An FDR < 0.05 and an absolute log2 fold change (|log2FC|) ≥ 0.5 were used as the criteria for identifying DEGs, ensuring consistency and comparability with the RNA-seq analyses.

### 2.5 CUT&Tag and ATAC-seq data processing and annotation

Publicly available ATAC-seq dataset GSE294915 and H3K4me1 CUT&Tag dataset GSE294916 were downloaded from the GEO database (35). Detailed information on these datasets is provided in **Supplementary Table 2**. The datasets deposited in GEO consisted of peak count matrices generated after peak calling by the original authors.

Both the ATAC-seq and CUT&Tag datasets were generated from two healthy donors, each subjected to three experimental conditions: Control, IFN-γ stimulation, and IFN-γ plus Ruxolitinib treatment, with two technical replicates for each condition. Because peak calling was performed independently for each donor, the resulting peak sets were not identical. Therefore, peak merging across donors, joint normalization, and genome-wide differential peak analysis were not performed. Instead, all downstream analyses were conducted separately for each donor.

For each donor, peak count matrices were normalized using the DESeq2 size factor method, followed by variance stabilizing transformation (VST) to obtain normalized peak signals for comparison across experimental conditions (31).

Unique peak identifiers were subsequently generated based on genomic coordinates. Peak annotation was performed using the ChIPseeker package together with the TxDb.Hsapiens.UCSC.hg38.knownGene and org.Hs.eg.db annotation databases (38). Promoter regions were defined as ±3 kb relative to the transcription start site (TSS). ATAC-seq and H3K4me1 CUT&Tag peaks annotated to the OAS3 promoter region were extracted, and the corresponding normalized peak signals, VST values, and genomic annotation information were obtained. The mean VST value across technical replicates was calculated for each experimental condition and used for data visualization.

Because the primary objective of this analysis was to characterize dynamic changes in chromatin accessibility and H3K4me1 enrichment at the OAS3 promoter following IFN-γ stimulation, data from the two donors were analyzed independently. Results from Subject 1 are presented as representative data, whereas those from Subject 2 were used to verify the reproducibility of the observed patterns. The results were interpreted descriptively without formal statistical inference (39, 40).

### 2.6 KEGG pathway enrichment analysis

Kyoto Encyclopedia of Genes and Genomes (KEGG) pathway enrichment analysis was performed using the clusterProfiler package in R (41). DEGs identified from each transcriptomic dataset were used as input for KEGG over-representation analysis (ORA) (42).

ORA was performed using the hypergeometric test to evaluate whether DEGs were significantly overrepresented in individual KEGG pathways. P values were adjusted for multiple testing using the BH procedure, and pathways with an FDR < 0.05 were considered significantly enriched.

For visualization, significantly enriched KEGG pathways containing at least one shared upregulated DEG were subsequently selected. The enrichment results were presented as combined bubble–Sankey plots, with the bubble plot on the left and the Sankey diagram on the right. The bubble plot summarizes the selected enriched KEGG pathways, whereas the Sankey diagram illustrates the associations between the shared upregulated DEGs and their corresponding enriched KEGG pathways.

### 2.7 Gene set enrichment analysis

To evaluate coordinated transcriptomic changes at the pathway level, GSEA was performed using the clusterProfiler package in R (41, 43).Gene symbols were mapped to Entrez Gene IDs using the org.Hs.eg.db annotation package. For each comparison, genes with valid Entrez Gene IDs were retained, and duplicated Entrez Gene IDs were resolved prior to construction of the ranked gene list. Genes were then ranked in descending order according to their log_2_FC, and KEGG GSEA was performed using the gseKEGG function without prior selection of differentially expressed genes.

Pathway enrichment was quantified using the normalized enrichment score (NES). Enrichment P values were calculated using the GSEA procedure implemented in clusterProfiler and adjusted for multiple testing using the BH procedure. Pathways with an FDR < 0.05 were considered significantly enriched.

Different visualization approaches were applied according to the analytical objectives. UpSet plots were used to illustrate the overlap of significantly enriched pathways among different comparisons, whereas ridge plots were used to visualize the distribution of leading-edge genes from representative KEGG pathways across the pre-ranked gene lists together with the corresponding NES values.

For the OAS3-knockout THP-1 dataset (GSE113363), the experimental design comprised one WT sample and two independent OAS3-KO clones. Therefore, GSEA was performed solely as an exploratory pathway-level analysis based on the ranked gene list. Although NES values, nominal P values, and BH-adjusted FDR values were generated by the GSEA algorithm, these statistics were used only to prioritize pathways within this dataset and were not interpreted as formal statistical inference because of the limited biological replication. Accordingly, the observed pathway alterations may reflect both OAS3 deficiency and clone-specific transcriptional differences.

### 2.8 Comparison of pathway enrichment between metabolic conditions (ΔNES analysis)

To compare differences in IFN-γ-induced pathway enrichment under distinct metabolic conditions, GSEA results from the Gal_IFNγ vs. Gal_Control and Glc_IFNγ vs. Glc_Control comparisons in the GSE290458 dataset were further analyzed. For KEGG pathways identified in both comparisons, the difference in normalized enrichment score (ΔNES) was calculated as follows:

ΔNES = NES(Gal_IFNγ vs. Gal_Control) − NES(Glc_IFNγ vs. Glc_Control)​

The ΔNES value was used as a descriptive metric to quantify differences in pathway enrichment between metabolic conditions and was not considered an independent statistical test. Pathways were classified as relatively enhanced under galactose (Gal) conditions when the corresponding pathway showed FDR < 0.05 in the Gal_IFNγ vs. Gal_Control comparison and ΔNES ≥ 0.5. Conversely, pathways were classified as relatively enhanced under glucose (Glc) conditions when the corresponding pathway showed FDR < 0.05 in the Glc_IFNγ vs. Glc_Control comparison and ΔNES ≤ −0.5. The results were visualized using ΔNES scatter plots.

### 2.9 Single-cell transcriptomic analysis

Publicly available scRNA-seq datasets GSE181279 and GSE157278 were downloaded from the GEO database (44, 45). Detailed information on these datasets is provided in **Supplementary Table 2**. Data preprocessing and downstream analyses were performed using the Seurat package (version 5.0) in R (46, 47).

For each cell, the number of detected genes (nFeature_RNA), total transcript counts (nCount_RNA), percentage of mitochondrial gene expression (percent.mt), and percentage of ribosomal gene expression (percent.rb) were calculated. Quality control thresholds were determined separately for each dataset according to the distributions of these metrics to remove low-quality cells, outlier cells, and potential doublets. Because sequencing depth and cellular composition differed between datasets, GSE181279 and GSE157278 were subjected to independent quality control criteria. The quality control workflow, filtering thresholds, numbers of retained cells, and quality assessment results are presented in **Supplementary Figures 1**, **S5**.

Following quality control, data were normalized using the LogNormalize method (scale.factor = 10,000). The top 2,000 highly variable genes were identified, and expression values were scaled using the ScaleData function. Principal component analysis (PCA) was subsequently performed, and batch effects were corrected using the Harmony algorithm (48). The optimal parameters for dimensionality reduction and clustering were determined for each dataset based on Elbow plots and Clustree analysis. Unsupervised cell clustering and Uniform Manifold Approximation and Projection (UMAP) visualization were then performed in the Harmony-corrected low-dimensional space. Details of parameter selection and clustering results are provided in **Supplementary Figures 2**, **S6**.

Cluster-specific marker genes were identified using the FindAllMarkers function (min.pct = 0.25, logfc.threshold = 0.25). Cell types were manually annotated based on canonical peripheral blood mononuclear cell (PBMC) marker genes reported in the literature (PMID: 40112801) (49). The resulting cell type annotations and marker gene expression profiles are shown in **Supplementary Figures 3**, **S7**.

### 2.10 IFN-γ module scoring

To quantify IFN-γ-related transcriptional activity at the single-cell level, the 315 IFN-γ-related genes integrated in Section 2.2 were used as the input gene set for module scoring. IFN-γ scores were calculated for each cell using the AddModuleScore function implemented in the Seurat package (46). The IFN-γ score represents the overall expression level of the IFN-γ-related gene set within an individual cell and was used to assess the IFN-γ-associated transcriptional response, rather than the expression level of IFN-γ itself.

The distribution of IFN-γ scores and IFN-γ-related transcriptional response patterns across different cell types were subsequently evaluated using UMAP visualization, violin plots, and candidate gene expression profiles. The corresponding results are presented in **Supplementary Figures 4**, **S8**.

### 2.11 Peripheral blood sample collection and PBMC isolation

The study protocol was approved by the Medical Ethics Committee of the First People’s Hospital of Jinzhong City (Approval No. KYLSZ [2026-034-01]), and written informed consent was obtained from all participants prior to sample collection. Peripheral venous blood samples were collected from healthy controls (HCs), patients with pSS, and patients with AD. Blood samples were collected into EDTA-containing anticoagulant tubes and processed promptly after collection.

PBMCs were isolated by Ficoll density gradient centrifugation. After isolation, PBMCs were washed with phosphate-buffered saline (PBS), counted, and subsequently used for protein extraction and Western blot analysis.

For the Western blot analysis comparing disease groups, the HCs, pSS, and AD groups each included three independent participants (n = 3), with each participant representing one independent biological replicate.

### 2.12 *In vitro* stimulation assay

Peripheral venous blood was collected from one healthy donor, and PBMCs were isolated by Ficoll density gradient centrifugation. PBMCs were cultured in RPMI-1640 medium supplemented with 10% fetal bovine serum (FBS) and 1% penicillin–streptomycin at 37 °C in a humidified incubator with 5% CO_2_.

Cells were stimulated with recombinant human IFN-γ (100 ng/mL) for 6 h or 24 h, while unstimulated cells served as the control. Following stimulation, cells were harvested for Western blot analysis.

PBMCs from a single healthy donor were used in this experiment. Each experimental condition was performed with three technical replicates, which were included solely to assess the reproducibility of protein expression changes and were not considered independent biological replicates for statistical analysis.

### 2.13 Western blot analysis

PBMCs were lysed in RIPA lysis buffer to extract total protein, and protein concentrations were determined using the bicinchoninic acid (BCA) assay. For each sample, 30 μg of total protein was separated by SDS–PAGE and subsequently transferred onto polyvinylidene difluoride (PVDF) membranes.

PVDF membranes were blocked with 5% non-fat milk or 5% bovine serum albumin (BSA) for 1 h at room temperature, followed by overnight incubation at 4 °C with the following primary antibodies: anti-OAS3 (Rabbit IgG, Cat. No. 85392-2-RR, Proteintech), anti-IRF5 (Mouse IgG1, Cat. No. 66835-1-PBS, Proteintech), and anti-GAPDH. After washing with TBST, membranes were incubated with horseradish peroxidase (HRP)-conjugated secondary antibodies for 1 h at room temperature. Protein bands were visualized using an enhanced chemiluminescence (ECL) detection system and imaged.

Band intensities were quantified using ImageJ software. The expression level of each target protein was normalized to the corresponding GAPDH signal (target protein/GAPDH). Relative protein expression was then calculated by setting the mean value of the HCs group to 1. Protein extraction, Western blot analysis, and densitometric quantification were performed independently for all samples. No pooled protein lysates were used in any of the analyses.

### 2.14 Statistical analysis

Bioinformatic analyses of the GEO datasets were performed using R (version 4.5.2). Statistical analyses and graphical visualization of the Western blot data were conducted using GraphPad Prism (version 9.5, GraphPad Software, San Diego, CA, USA).

All data are presented as the mean ± standard deviation (SD). For the Western blot analyses of clinical samples, each individual participant was treated as an independent biological replicate. Technical replicates were used solely to assess experimental reproducibility, and their mean value was used as the final measurement for the corresponding biological sample; technical replicates were not considered independent observations for statistical analysis. Comparisons among groups were performed using ordinary one-way analysis of variance (ANOVA) followed by Tukey’s multiple comparisons test for pairwise comparisons. All statistical tests were two-tailed, and P < 0.05 was considered statistically significant.

For the *in vitro* IFN-γ stimulation experiment, PBMCs from a single healthy donor were used, with three technical replicates for each treatment condition. As no independent biological replicates were included, these data were used only to illustrate the trend of protein expression changes, and no inferential statistical analyses were performed.

## 3 Results

### 3.1 Identification of shared IFN-γ-related genes between AD and pSS

**Figure 2:**
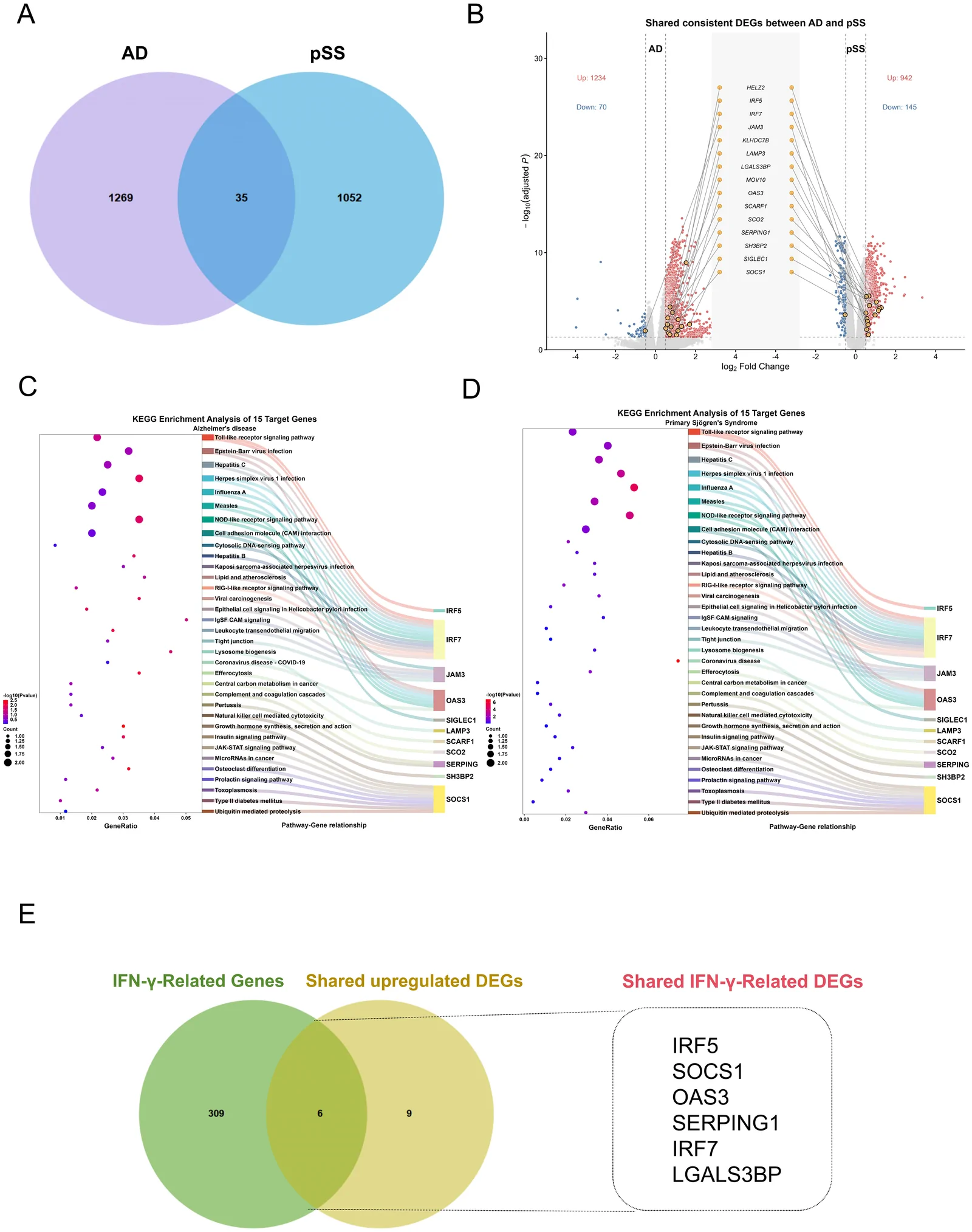
Identification of shared IFN-γ-related genes between AD and pSS. **(A)** Venn diagram illustrating the overlap of DEGs between AD and pSS. Purple represents AD, blue represents pSS, and the overlapping region indicates the shared DEGs. **(B)** Volcano plots showing DEGs in AD (left) and pSS (right). Red dots indicate upregulated genes, whereas blue dots indicate downregulated genes. Labeled genes represent the shared DEGs exhibiting consistent directions of differential expression in both diseases. **(C)** Bubble–Sankey plot illustrating the KEGG pathway enrichment profile associated with the 15 shared upregulated DEGs in AD. The left panel presents the KEGG pathway enrichment results generated from all DEGs identified in AD, with only significantly enriched pathways containing at least one shared upregulated DEG retained for visualization. Bubble size represents the number of enriched genes, and bubble color indicates the statistical significance of pathway enrichment. The right Sankey diagram illustrates the associations between the shared upregulated DEGs and their corresponding enriched KEGG pathways. **(D)** Bubble–Sankey plot illustrating the KEGG pathway enrichment profile associated with the 15 shared upregulated DEGs in pSS. The visualization is presented in the same format as in **(C)**, using KEGG enrichment results generated from all DEGs identified in pSS. **(E)** Venn diagram showing the overlap between IFN-γ-related genes and the shared upregulated DEGs. Green represents IFN-γ-related genes, yellow represents the shared upregulated DEGs, and the overlapping region identifies the shared IFN-γ-related DEGs, including IRF5, SOCS1, OAS3, SERPING1, IRF7, and LGALS3BP.

To investigate the potential mechanisms underlying the comorbidity of AD and pSS, two publicly available PBMC transcriptomic datasets were analyzed, including GSE248417 (49 patients with AD and 49 healthy controls) and GSE84844 (30 patients with pSS and 30 healthy controls) (**Supplementary Table 2**). Differential expression analysis was performed independently for each dataset, and the overlap between the two sets of DEGs was determined. A total of 35 common DEGs were identified (**Figure 2A**; **Supplementary Table 3**), among which 15 genes were significantly upregulated in both AD and pSS (log2FC > 0.5, FDR < 0.05) (**Figure 2B**).

Disease-specific KEGG pathway enrichment analysis was first performed independently using the complete DEG sets identified in the AD and pSS datasets. To characterize the functional context of the 15 shared upregulated DEGs, the resulting KEGG enrichment profiles were subsequently filtered to retain pathways containing at least one of these shared genes. The selected pathways were predominantly associated with innate immunity, antiviral responses, and viral infection, including the Toll-like receptor signaling pathway, RIG-I-like receptor signaling pathway, cytosolic DNA-sensing pathway, Epstein–Barr virus infection, Influenza A, Herpes simplex virus 1 infection, and Hepatitis C. Interferon-related immune pathways were consistently represented in both disease-specific enrichment profiles (**Figures 2C, D**; **Supplementary Table 4**).

To further focus on IFN-γ-related mechanisms, the 15 shared upregulated DEGs were intersected with the predefined IFN-γ-related gene set, resulting in the identification of six shared IFN-γ-related DEGs (**Figure 2E**).

Collectively, these findings identified six shared IFN-γ-related candidate genes between AD and pSS, providing the basis for the subsequent single-cell and experimental analyses.

### 3.2 Single-cell transcriptomic analysis reveals shared IFN-γ-responsive monocyte subsets in AD and pSS

Following the identification of shared IFN-γ-related candidate genes, scRNA-seq data from PBMCs were analyzed to determine their predominant cellular sources.

**Figure 3:**
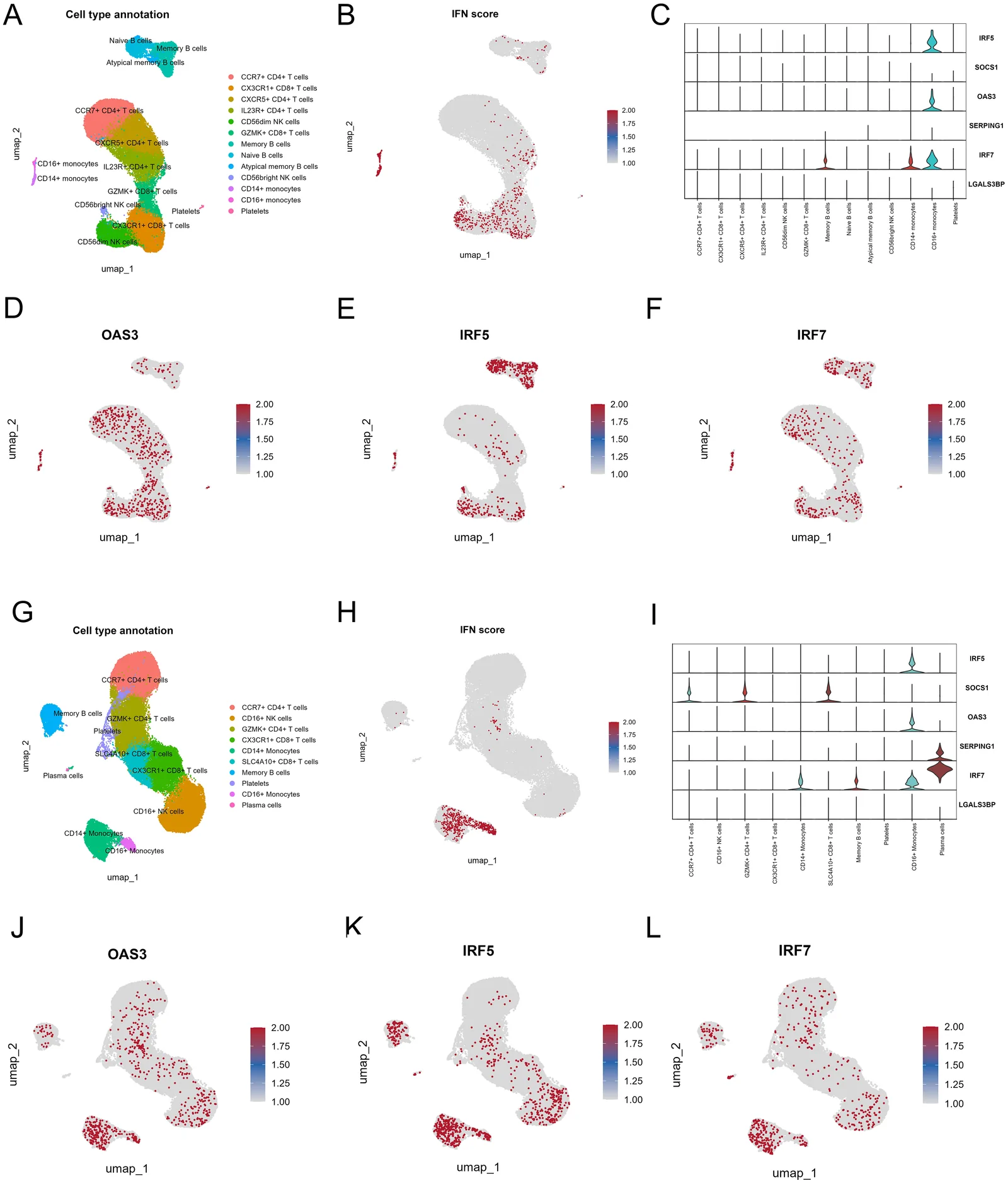
Single-cell transcriptomic analyses reveal IFN-γ-responsive monocytes and the expression patterns of candidate genes in AD and pSS. **(A)** UMAP plot showing the distribution of peripheral blood cell clusters and cell type annotations in AD. **(B)** UMAP plot showing the distribution of IFN-γ response scores across cell clusters in AD, with red indicating higher IFN-γ response scores. **(C)** Violin plots showing the expression distributions of the candidate genes (IRF5, SOCS1, OAS3, SERPING1, IRF7, and LGALS3BP) across different cell types in AD. **(D)** UMAP plot showing the expression pattern of OAS3 in AD.**(E)** UMAP plot showing the expression pattern of IRF5 in AD. **(F)** UMAP plot showing the expression pattern of IRF7 in AD. **(G)** UMAP plot showing the distribution of peripheral blood cell clusters and cell type annotations in pSS. **(H)** UMAP plot showing the distribution of IFN-γ response scores across cell clusters in pSS. **(I)** Violin plots showing the expression distributions of the candidate genes across different cell types in pSS.**(J)** UMAP plot showing the expression pattern of OAS3 in pSS. **(K)** UMAP plot showing the expression pattern of IRF5 in pSS. **(L)** UMAP plot showing the expression pattern of IRF7 in pSS.

In the AD PBMC scRNA-seq dataset GSE181279 (3 patients with AD and 2 healthy controls) (**Supplementary Table 2**), 13 distinct cell populations were identified after cell type annotation based on canonical marker genes (**Figure 3A**; **Supplementary Figures 1**-**S3**). Calculation of the IFN-γ score revealed that CD16^+^ monocytes exhibited the highest IFN-γ score, followed by CD14^+^ monocytes (**Figure 3B**; **Supplementary Figure 4**), indicating that monocytes displayed the strongest IFN-γ-associated transcriptional response. Expression profiling of the six shared IFN-γ-related DEGs further demonstrated that IRF5, OAS3, and IRF7 were most highly expressed in CD16^+^ monocytes (**Figure 3C**). FeaturePlot analysis showed that all three genes were predominantly enriched in the monocyte compartment and were detectable in both CD16^+^ and CD14^+^ monocytes, with a broader distribution of positive cells observed in the CD16^+^ monocyte population (**Figures 3D–F**; **Supplementary Figure 4**).

In the pSS PBMC scRNA-seq dataset GSE157278 (5 patients with pSS and 5 healthy controls) (**Supplementary Table 2**), nine cell populations were identified (**Figure 3G**; **Supplementary Figures 5**-**S7**). Consistent with the findings in AD, CD16^+^ monocytes exhibited the highest IFN-γ score, followed by CD14^+^ monocytes (**Figure 3H**; **Supplementary Figure 8**). Similarly, IRF5, OAS3, and IRF7 showed the highest expression levels in CD16^+^ monocytes (**Figure 3I**). FeaturePlot analysis demonstrated that all three genes were predominantly expressed in monocytes and were detectable in both CD16^+^ and CD14^+^ monocyte subsets. In addition, IRF7 also exhibited relatively high and concentrated expression in plasma cells (**Figures 3J–L**; **Supplementary Figure 8**).

Collectively, these findings indicate that CD16^+^ and CD14^+^ monocytes are the major IFN-γ-responsive cell populations in the peripheral blood of both AD and pSS. Among the candidate genes, IRF5 and OAS3 were predominantly enriched in monocytes, with the highest expression observed in CD16^+^ monocytes, whereas IRF7 displayed a broader cellular distribution, including prominent expression in plasma cells from patients with pSS. Accordingly, subsequent analyses focused on IRF5 and OAS3, which exhibited more pronounced monocyte-specific expression patterns.

### 3.3 OAS3 exhibits a robust IFN-γ-responsive expression pattern in the monocyte–macrophage lineage

**Figure 4:**
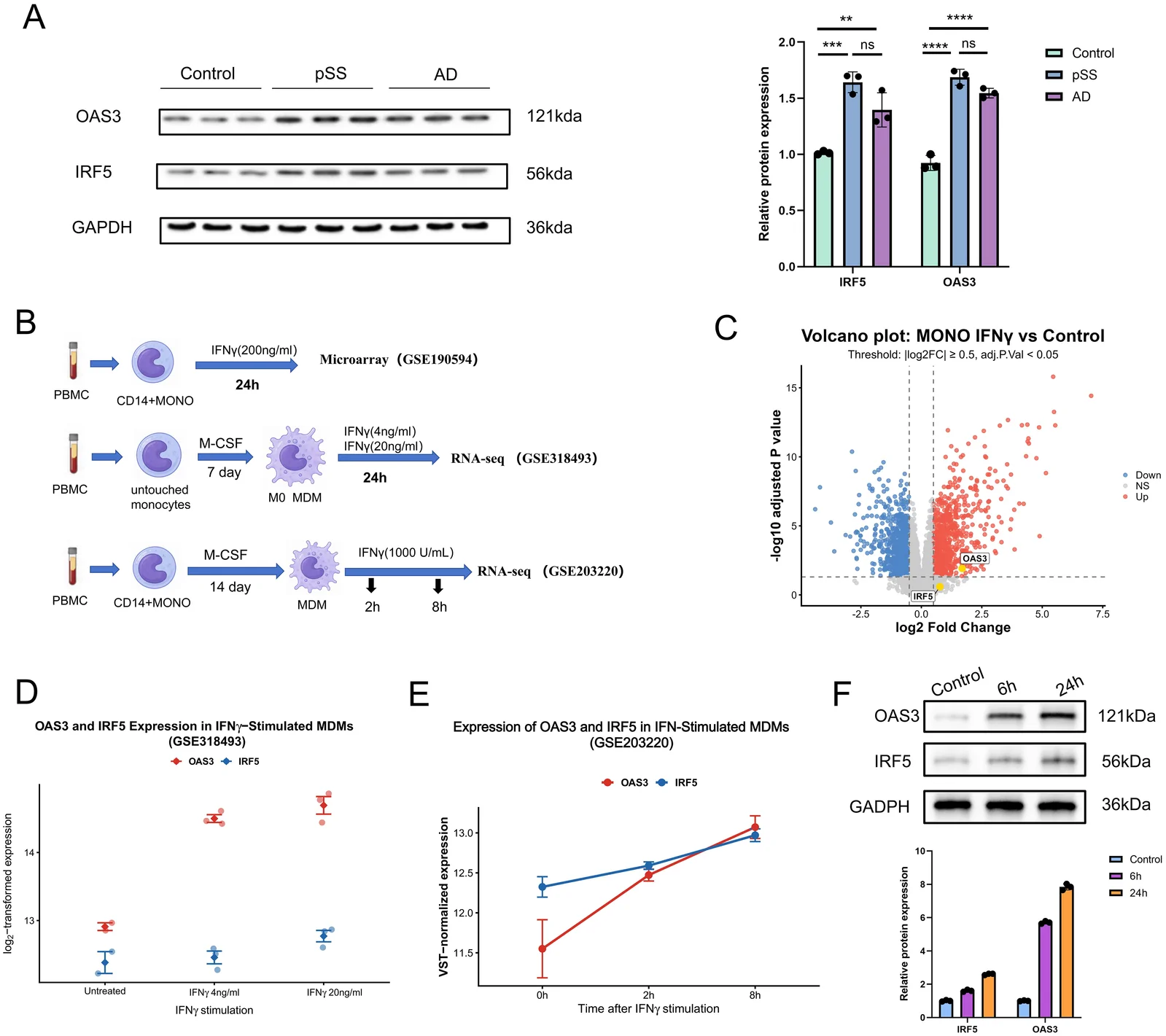
IFN-γ-induced expression of OAS3 and IRF5 in monocytes and macrophages. **(A)** Western blot analysis of IRF5 and OAS3 protein expression in PBMCs isolated from healthy controls (Control), patients with pSS, and patients with AD. Representative immunoblots are shown on the left, and densitometric quantification is presented on the right. Protein expression levels were first normalized to the corresponding GAPDH signal and subsequently normalized to the mean expression level of the Control group, which was set to 1. Each group included three independent biological replicates (independent donors, n = 3). For each biological sample, three technical replicates were performed to assess experimental reproducibility, and their average value was used as the final measurement for the corresponding biological sample. Statistical analyses were performed using biological replicates only. Data are presented as mean ± SD. Group comparisons were conducted using ordinary one-way analysis of variance (ANOVA) followed by Tukey’s multiple comparisons test. P < 0.05, P < 0.01, P < 0.001, P < 0.0001. **(B)** Schematic overview of the cross-dataset comparative analysis. Three independent GEO datasets were integrated to compare the expression responses of OAS3 and IRF5 under different IFN-γ stimulation conditions. GSE190594 analyzed PBMC-derived CD14^+^ monocytes stimulated with IFN-γ (200 ng/mL) for 24 h using microarray analysis. GSE318493 analyzed macrophage colony-stimulating factor (M-CSF)-derived human MDMs stimulated with IFN-γ (4 or 20 ng/mL) for 24 h using RNA sequencing. GSE203220 analyzed MDMs stimulated with IFN-γ (1000 U/mL) for 2 h and 8 h using RNA-seq. This schematic summarizes the integrated analytical design across independent public datasets with different cell types, stimulation doses, and treatment durations. Each dataset was analyzed independently and used solely to compare the expression responses of OAS3 and IRF5 under distinct IFN-γ stimulation conditions rather than to represent a continuous biological process. **(C)** Volcano plot showing DEGs in IFN-γ-treated monocytes from GSE190594. Red dots indicate upregulated genes, blue dots indicate downregulated genes, and OAS3 and IRF5 are highlighted in yellow. **(D)** Expression profiles of OAS3 and IRF5 in M-CSFs differentiated MDMs stimulated with two concentrations of IFN-γ (4 and 20 ng/mL) for 24 h (GSE318493). Red lines represent OAS3, and blue lines represent IRF5. **(E)** Time-dependent expression profiles of OAS3 and IRF5 in MDMs stimulated with IFN-γ for 2 h and 8 h (GSE203220). Red lines represent OAS3, and blue lines represent IRF5. **(F)** Western blot analysis of IRF5 and OAS3 protein expression in PBMCs obtained from a single healthy donor following IFN-γ stimulation (100 ng/mL) for 6 h and 24 h. Representative immunoblots are shown above, and densitometric quantification is presented below. Protein expression levels were normalized to the corresponding GAPDH signal and then normalized to the untreated Control, which was set to 1. PBMCs from one healthy donor were analyzed, with three technical replicates performed for each treatment condition. Technical replicates were used exclusively to assess experimental reproducibility, and their average values are presented to illustrate the temporal changes in protein expression. Because independent biological replicates were not included, no inferential statistical analysis was performed.

The preceding single-cell transcriptomic analyses showed that OAS3 and IRF5 were predominantly expressed in CD14^+^ and CD16^+^ monocytes, the two cell populations exhibiting the strongest IFN-γ-associated transcriptional responses in both AD and pSS. In addition, OAS3 was significantly upregulated in the two independent PBMC transcriptomic datasets (AD: log_2_FC = 1.29, FDR = 0.0039; pSS: log_2_FC = 0.97, FDR = 2.62 × 10^-^^4^). Based on these findings, Western blot analysis was performed to examine the protein expression of OAS3 and IRF5 in PBMCs from healthy controls, patients with pSS, and patients with AD. Compared with healthy controls, IRF5 protein expression was significantly increased in the pSS group (P < 0.001) and modestly increased in the AD group (P < 0.05). In contrast, OAS3 protein expression was markedly elevated in both AD and pSS patients (both P < 0.0001) (**Figure 4A**).

To further compare the IFN-γ responsiveness of OAS3 and IRF5, transcriptomic data from three independent GEO datasets were integrated (**Figure 4B**; **Supplementary Table 2**). In the monocyte dataset GSE190594 (IFN-γ group, n = 9; control group, n = 8), OAS3 was significantly upregulated following IFN-γ stimulation (log2FC = 1.688, FDR = 0.0122), whereas IRF5 showed no significant change (**Figure 4C**; **Supplementary Table 3**).

Next, transcriptomic data from monocyte-derived macrophages (MDMs) stimulated with different concentrations of IFN-γ (GSE318493; PBS control, n = 2; IFN-γ 4 ng/mL, n = 3; IFN-γ 20 ng/mL, n = 3) were analyzed. OAS3 expression was significantly induced by 4 ng/mL IFN-γ, whereas stimulation with 20 ng/mL did not produce a further substantial increase. In contrast, IRF5 expression exhibited only minimal changes (**Figure 4D**; **Supplementary Table 3**), indicating that OAS3 induction was not dose-dependent and could be robustly elicited by low-dose IFN-γ stimulation.

The transcriptional response of MDMs to different durations of IFN-γ stimulation was further examined using GSE203220 (unstimulated, n = 3; IFN-γ 2 h, n = 3; IFN-γ 8 h, n = 3). Expression of both OAS3 and IRF5 increased progressively with prolonged IFN-γ stimulation, with OAS3 exhibiting a more pronounced induction (**Figure 4E**), showing a progressive increase across the examined stimulation durations. Consistently, in an *in vitro* stimulation experiment using PBMCs from a healthy donor, OAS3 protein expression increased after 6 h of IFN-γ stimulation and was further elevated at 24 h, whereas IRF5 protein expression remained largely unchanged (**Figure 4F**), in agreement with the transcriptomic findings.

Collectively, these results demonstrate that OAS3 is a highly IFN-γ-responsive gene in the monocyte–macrophage lineage. Its expression exhibits a clear time-dependent induction but no apparent dose dependence, with robust upregulation achieved even under low-dose IFN-γ stimulation.

### 3.4 IFN-γ-associated inflammatory transcriptional responses differ between glucose and galactose culture conditions

**Figure 5:**
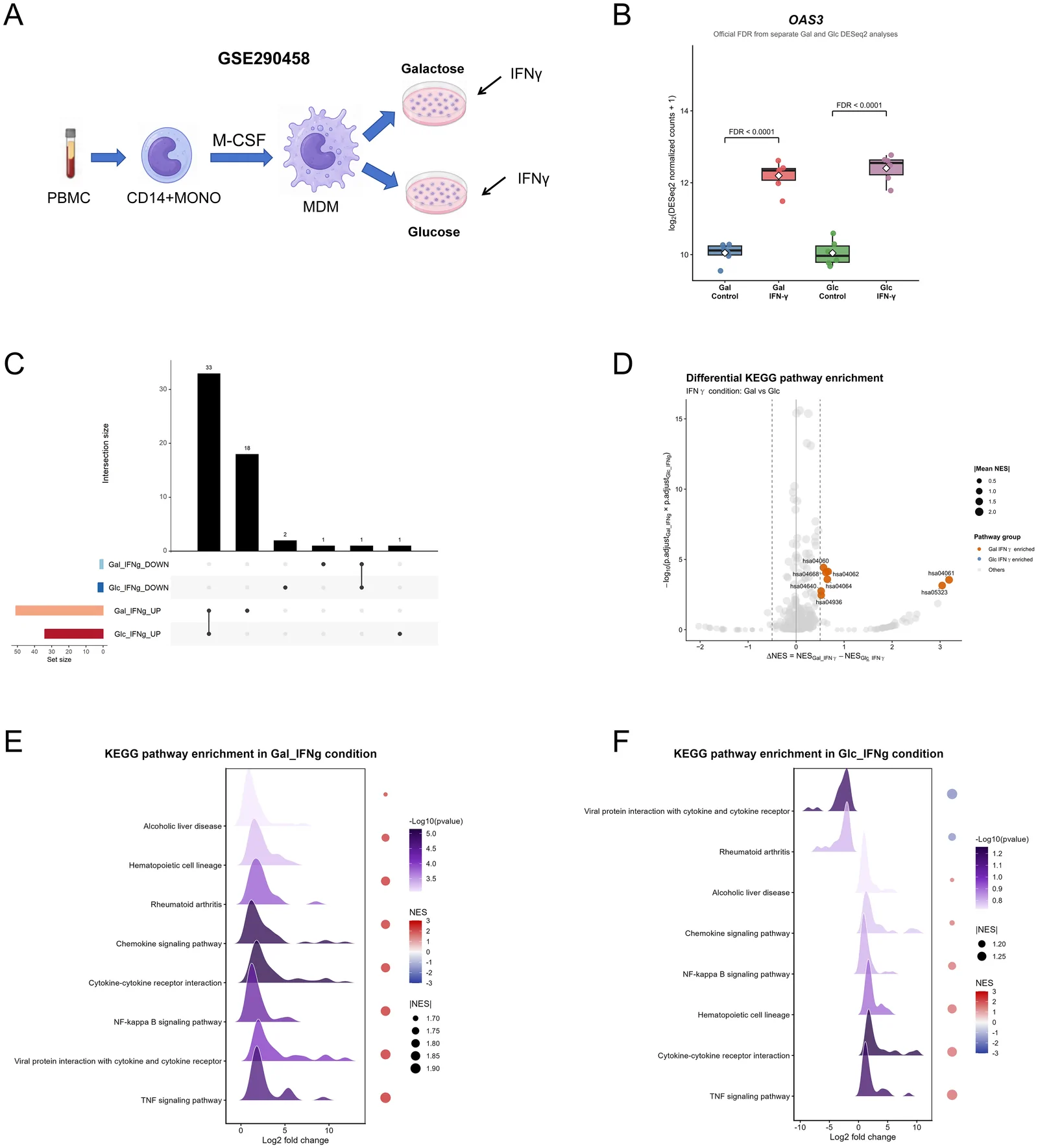
Metabolic state modulates the transcriptional response of macrophages to IFN-γ stimulation. **(A)** Schematic overview of the experimental design illustrating IFN-γ stimulation of MDMs under distinct metabolic conditions using a public GEO dataset. PBMC-derived CD14^+^ monocytes were differentiated into MDMs with M-CSF and cultured in either Glc or Gal medium before treatment with IFN-γ or vehicle control (Gal-Control, n = 6; Gal-IFNγ, n = 6; Glc-Control, n = 6; Glc-IFNγ, n = 6). The resulting transcriptomic data were used for differential expression and pathway enrichment analyses. **(B)** Box plots showing the DESeq2-normalized expression of OAS3 (log_2_ normalized counts + 1) in control and IFN-γ-treated MDMs cultured under Gal and Glc conditions. Each dot represents one biological replicate. Boxes indicate the interquartile range (IQR), the horizontal line denotes the median, and the white diamond indicates the mean. FDR values were obtained directly from the independent DESeq2 differential expression analyses performed under the Gal and Glc conditions. **(C)** UpSet plot showing the overlap of significantly enriched pathways identified by gene set enrichment analysis (GSEA; FDR < 0.05) following IFN-γ stimulation of MDMs under Gal and Glc culture conditions. **(D)** Scatter plot comparing GSEA pathway enrichment between Gal and Glc conditions following IFN-γ stimulation. ΔNES was calculated as: ΔNES = NES_(Gal_IFNγ vs Gal_Control) − NES_(Glc_IFNγ vs Glc_Control). Red dots indicate pathways preferentially enriched under Gal conditions (ΔNES ≥ 0.5 and FDR_GAL < 0.05), whereas blue dots indicate pathways preferentially enriched under Glc conditions (ΔNES ≤ −0.5 and FDR_GLC < 0.05). **(E)** GSEA ridge plot showing the enrichment profiles of the eight pathways significantly enhanced under Gal conditions, defined as ΔNES ≥ 0.5 and FDR_GAL < 0.05 [corresponding to the red dots in **(D)**]. **(F)** GSEA ridge plot showing the enrichment profiles of the same eight pathways under Glc conditions.

Previous studies have demonstrated that metabolic reprogramming plays a critical role in regulating macrophage responses to inflammatory stimuli (50). To further evaluate whether metabolic conditions influence the transcriptional response to IFN-γ, we analyzed the publicly available RNA-seq dataset GSE290458, comparing gene expression profiles following IFN-γ stimulation under Glc and Gal culture conditions. The analysis included four experimental groups: Gal_Control (n = 6), Gal_IFNγ (n = 6), Glc_Control (n = 6), and Glc_IFNγ (n = 6) (**Figure 5A**).

We first examined OAS3 expression under the two culture conditions. Differential expression analysis showed that IFN-γ robustly induced OAS3 expression under both glucose and galactose conditions. Specifically, OAS3 expression was significantly upregulated under Glc conditions (log_2_FC = 2.362, FDR = 3.09 × 10^-^²^8^) and was similarly increased under Gal conditions (log_2_FC = 2.170, FDR = 1.06 × 10^-^²^7^) (**Figure 5B**).

To further assess whether metabolic conditions influenced the overall IFN-γ-induced transcriptional response, GSEA was performed independently for the Gal_IFNγ vs. Gal_Control and Glc_IFNγ vs. Glc_Control comparisons. Across the two comparisons, 34 KEGG pathways were significantly enriched (FDR < 0.05) (**Figure 5C**; **Supplementary Table 5**). To quantify differences in pathway enrichment between the two culture conditions, we calculated the difference in normalized enrichment scores (ΔNES = NES_Gal−IFNγ − NES_Glc−IFNγ). Eight pathways met the predefined criteria (ΔNES ≥ 0.5 and FDR_Gal < 0.05)and were therefore classified as showing relatively stronger enrichment under galactose than under glucose culture conditions (**Figure 5D**).

The pathways classified as showing relatively stronger enrichment under galactose conditions were predominantly related to canonical inflammatory signaling pathways, including the NF-κB signaling pathway, TNF signaling pathway, Chemokine signaling pathway, and Cytokine–cytokine receptor interaction (**Figures 5E, F**; **Supplementary Table 7**). In addition, these pathways were mainly associated with inflammatory cytokine and chemokine signaling.

Collectively, these findings indicate that IFN-γ consistently induces OAS3 expression under both glucose and galactose culture conditions. Comparative GSEA showed that several inflammation-related pathways exhibited relatively stronger enrichment under galactose than under glucose culture conditions.

### 3.5 IFN-γ-induced OAS3 expression is associated with epigenetic remodeling

**Figure 6:**
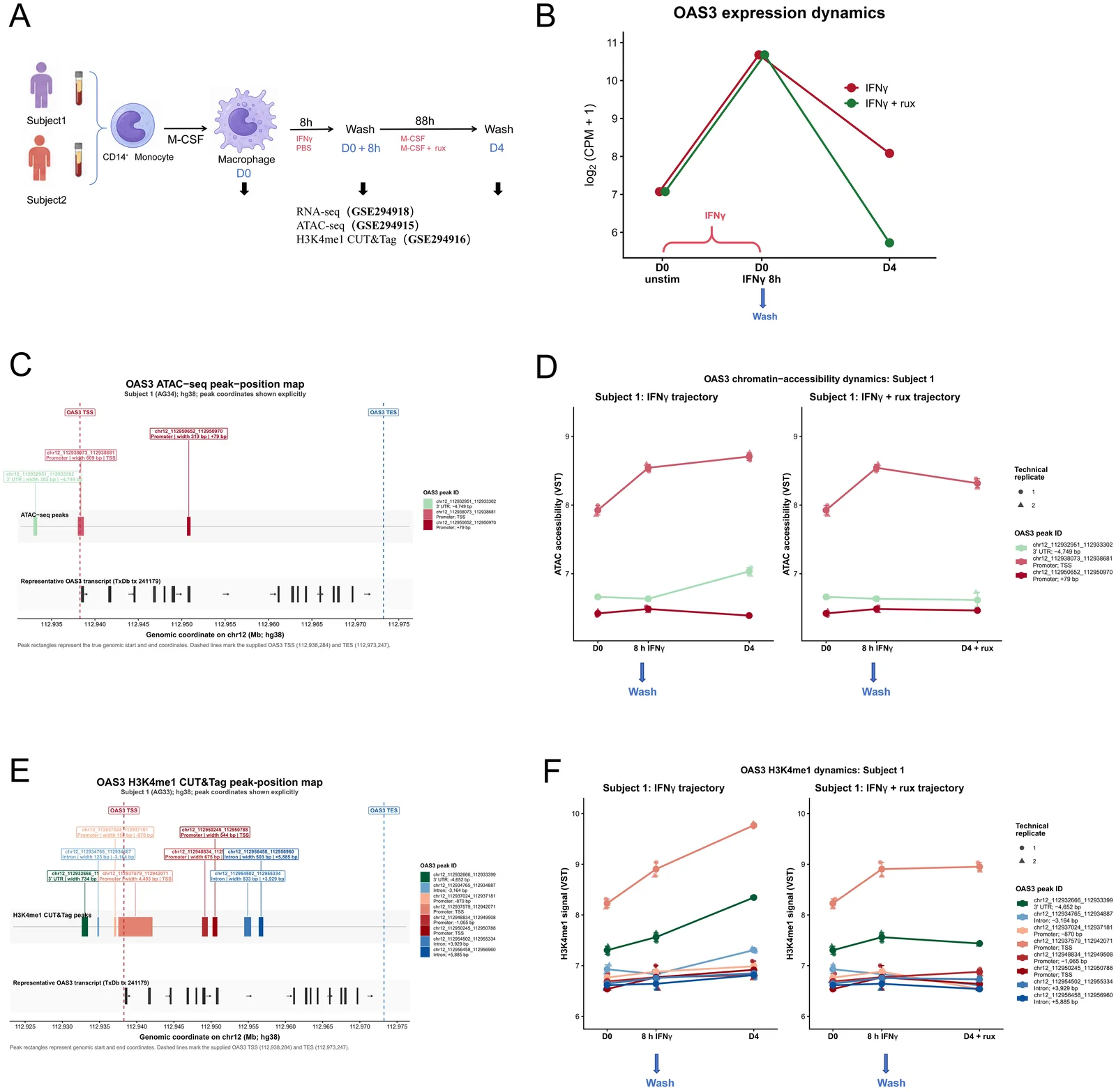
IFN-γ-induced epigenetic remodeling at the OAS3 locus. **(A)** Schematic overview of the experimental design based on a publicly available dataset (PMID: 41706052). PBMC-derived CD14^+^ monocytes were differentiated into macrophages (Day 0, D0) using M-CSF, followed by IFN-γ stimulation for 8 h. After IFN-γ washout, cells were cultured for an additional 88 h and subsequently treated with either M-CSF alone (D4 IFN-γ) or M-CSF plus the JAK inhibitor ruxolitinib (D4 IFN-γ + rux). Integrated analyses were performed using RNA sequencing (RNA-seq; GSE294918), ATAC sequencing (ATAC-seq; GSE294915), and H3K4me1 CUT&Tag (GSE294916) datasets. The RNA-seq dataset was generated from one healthy donor, whereas the ATAC-seq and H3K4me1 CUT&Tag datasets were generated from two healthy donors, with two technical replicates included for each treatment condition. **(B)** Dynamic expression profile of OAS3 determined by RNA-seq under the indicated treatment conditions. **(C)** Genomic distribution of ATAC-seq peaks at the OAS3 locus in Subject 1 (hg38, chromosome 12). Colored rectangles indicate the genomic positions and spans of ATAC-seq peaks, corresponding to promoter (red), 3′ untranslated region (3′ UTR; green), intronic (blue), and other (gray-purple) genomic regions. Representative OAS3 transcript models and exon structures are shown below. Arrows indicate the direction of transcription, and dashed lines denote the transcription start site (TSS) and transcription end site (TES). **(D)** Dynamic changes in chromatin accessibility at OAS3-associated ATAC-seq peaks in Subject 1 across different treatment conditions. Each line represents an individual ATAC-seq peak. Data points represent the mean of two technical replicates, and error bars indicate variation between technical replicates. **(E)** Genomic distribution of H3K4me1 CUT&Tag peaks at the OAS3 locus in Subject 1. Colored rectangles indicate the genomic positions and spans of H3K4me1 peaks, corresponding to promoter (red), 3′ untranslated region (3′ UTR; green), intronic (blue), and other (gray-purple) genomic regions. Representative OAS3 transcript models and exon structures are shown below. Arrows indicate the direction of transcription, and dashed lines denote the TSS and TES. **(F)** Dynamic changes in H3K4me1 enrichment at OAS3-associated peaks in Subject 1 across different treatment conditions. Each line represents an individual H3K4me1 peak. Data points represent the mean of two technical replicates, and error bars indicate variation between technical replicates.

The preceding analyses demonstrated that OAS3 responds robustly to IFN-γ stimulation across different stimulation durations, cytokine concentrations, and metabolic conditions. To further investigate the transcriptional regulatory changes accompanying IFN-γ-induced OAS3 expression, we integrated RNA-seq (GSE294918), ATAC-seq (GSE294915), and H3K4me1 CUT&Tag (GSE294916) datasets from the same published study (PMID: 41706052). These datasets were generated using a unified experimental design in which PBMC-derived CD14^+^ monocytes were differentiated into macrophages with M-CSF, followed by IFN-γ stimulation, stimulus withdrawal, or treatment with the JAK inhibitor ruxolitinib, after which multi-omics profiling was performed (**Figure 6A**; **Supplementary Table 2**).

RNA-seq analysis (GSE294918; one healthy donor) showed that OAS3 expression increased rapidly after 8 h of IFN-γ stimulation. OAS3 expression subsequently declined following IFN-γ withdrawal, whereas treatment with ruxolitinib further suppressed its expression (**Figure 6B**).

ATAC-seq analysis (GSE294915; two healthy donors, with two technical replicates per experimental condition) identified three accessible chromatin peaks within the OAS3 locus in Subject 1, including two promoter-associated peaks and one intronic peak (**Figure 6C**). Dynamic analysis revealed that the major promoter-associated peak exhibited markedly increased chromatin accessibility after 8 h of IFN-γ stimulation and remained highly accessible following stimulus withdrawal. In contrast, chromatin accessibility at this peak was reduced after ruxolitinib treatment compared with the stimulus-withdrawal condition. The remaining two peaks showed only modest changes across experimental conditions (**Figure 6D**; **Supplementary Table 6**).

To further evaluate promoter activation, changes in H3K4me1 occupancy across the OAS3 locus were examined. CUT&Tag analysis (GSE294916; two healthy donors, with two technical replicates per experimental condition) detected multiple H3K4me1-enriched peaks, including peaks located within the promoter and intronic regions (**Figure 6E**; **Supplementary Table 6**). Consistent with the ATAC-seq results, the promoter-associated H3K4me1 signal increased markedly following IFN-γ stimulation and remained elevated after stimulus withdrawal, whereas ruxolitinib treatment substantially attenuated this enrichment. In contrast, changes in the remaining peaks were relatively limited (**Figure 6F**; **Supplementary Table 6**).

Independent analysis of the second healthy donor (Subject 2) revealed dynamic changes in ATAC-seq and H3K4me1 CUT&Tag signals across the OAS3 locus that were consistent with those observed in Subject 1 (**Supplementary Figure 9**; **Supplementary Table 6**), providing additional descriptive support for these findings.

Collectively, these results demonstrate that IFN-γ-induced upregulation of OAS3 is accompanied by increased chromatin accessibility and enhanced H3K4me1 enrichment at its promoter region. These epigenetic changes were markedly attenuated by ruxolitinib treatment, suggesting that IFN-γ-induced transcriptional activation of OAS3 is accompanied by dynamic epigenetic remodeling.

### 3.6 OAS3 deficiency is associated with altered immune- and neurodegeneration-related transcriptional programs

**Figure 7:**
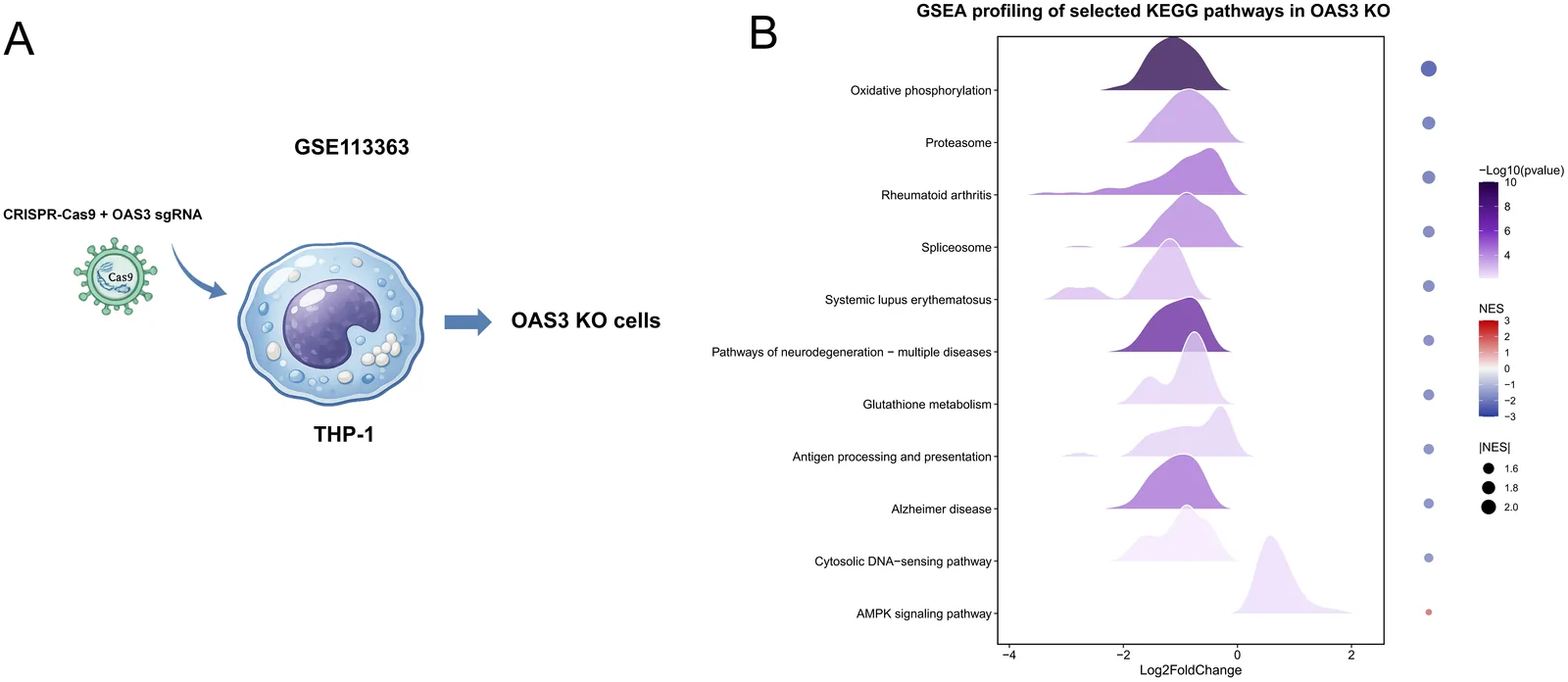
OAS3 deficiency is associated with transcriptional alterations in immune- and neurodegeneration-related pathways. **(A)** Schematic overview of the experimental design using the GEO dataset GSE113363. OAS3-KO THP-1 cells were generated using the CRISPR/Cas9 system. Under unstimulated conditions (No), RNA sequencing data from WT THP-1 cells (n = 1) were compared with those from two independent OAS3-KO clones (n = 2) to explore transcriptional changes associated with OAS3 deficiency under basal conditions. **(B)** GSEA ridge plot illustrating the KEGG pathway enrichment profile associated with OAS3 knockout, providing a preliminary exploration of the potential biological functions of OAS3. Representative pathways related to neurodegenerative diseases, immune and inflammatory responses, and energy metabolism are shown. Ridge curves represent the distribution of log_2_ fold changes of core enrichment genes within each pathway, and ridge fill intensity indicates the nominal enrichment significance (−log_10_ P value). The color of the dot on the right represents the normalized enrichment score (NES), with deeper blue indicating stronger negative enrichment and deeper red indicating stronger positive enrichment. Dot size is proportional to the absolute value of the NES (|NES|).

To further explore the potential biological functions of OAS3, we analyzed the publicly available RNA-seq dataset GSE113363, comparing the transcriptomic profiles of unstimulated WT THP-1 cells (n = 1) with those of two independent OAS3-KO THP-1 cell clones (n = 2) (**Figure 7A**; **Supplementary Table 2**). Given the limited sample size and the inclusion of independent knockout clones, GSEA was performed as an exploratory pathway-level analysis, and the results were interpreted descriptively.

Exploratory GSEA identified 59 KEGG pathways that met the algorithm-defined significance threshold (FDR < 0.05). Among these, several neurodegeneration-related pathways showed negative enrichment, including Alzheimer disease (NES = −1.53), Parkinson disease (NES = −1.98), Huntington disease (NES = −1.79), and Pathways of neurodegeneration – multiple diseases (NES = −1.58). In addition, pathways involved in mitochondrial energy metabolism, including Oxidative phosphorylation, together with several immune-related pathways, such as Rheumatoid arthritis, Systemic lupus erythematosus, Antigen processing and presentation, and the Cytosolic DNA-sensing pathway, also exhibited negative enrichment (**Figure 7B**; **Supplementary Table 5**).

Overall, these exploratory analyses suggest that transcriptional alterations associated with OAS3 deficiency are accompanied by reduced enrichment of multiple immune-inflammatory and neurodegeneration-related pathways. Owing to the limited biological replication in this dataset, these findings should be regarded as hypothesis-generating rather than confirmatory evidence. The reported enrichment statistics were used to prioritize pathways for biological interpretation and should not be interpreted as formal statistical inference.

## 4 Discussion

Previous epidemiological studies have demonstrated that patients with pSS are at a significantly increased risk of developing AD, suggesting that these two disorders may share common immunopathological mechanisms. In the present study, by integrating multi-omics analyses with experimental validation, we identified a shared IFN-γ-associated immune signature between AD and pSS, which was predominantly localized to CD14^+^ and CD16^+^ monocytes. Furthermore, we identified OAS3 as a candidate IFN-γ-responsive downstream molecule that exhibited sustained expression across the examined datasets and experimental conditions. These findings suggest that OAS3 may represent a shared peripheral immune feature of pSS and AD and provide a basis for further investigation of its potential role in IFN-γ-associated inflammatory processes.

First, integrative multi-omics analyses identified a set of shared IFN-γ-related DEGs in the peripheral blood of patients with AD and pSS. Notably, this transcriptional signature was predominantly enriched in CD14^+^ and CD16^+^ monocytes, with OAS3 and IRF5 emerging as two of the most prominently enriched genes. Western blot analysis further confirmed that the protein expression levels of both OAS3 and IRF5 were significantly elevated in PBMCs from patients with AD and pSS. Previous studies have established that monocytes and their differentiated macrophages are major effector cells mediating IFN-γ-driven innate immune responses (51–53). Moreover, persistent monocyte activation and chronic inflammatory dysregulation have been reported in both pSS and AD (54–57). Taken together with our findings, these observations are consistent with the possibility that IFN-γ-associated monocyte activation represents a shared peripheral immune feature of pSS and AD. However, whether this shared immune feature contributes to their epidemiological association remains to be determined.

Based on the shared IFN-γ-related transcriptional signatures, we further investigated key effector molecules that may contribute to the sustained inflammatory response. Although both OAS3 and IRF5 were enriched in monocytes from patients with AD and pSS, they exhibited distinct responses to IFN-γ stimulation. Integrated analysis of multiple GEO datasets showed that OAS3 was rapidly induced by IFN-γ and displayed sustained, time-dependent expression, whereas IRF5 exhibited relatively limited transcriptional changes. Previous studies have identified OAS3 as a classical ISG that is rapidly induced by IFN-γ (58, 59). In contrast, IRF5 primarily functions as an upstream regulator within IFN-associated inflammatory signaling networks (60–65). Therefore, compared with IRF5, OAS3 is more likely to serve as a candidate marker of sustained IFN-γ transcriptional activity in monocytes/macrophages.

Because immune cell responses to IFN-γ are influenced not only by cytokine stimulation but also by cellular metabolism, we further investigated whether different metabolic conditions affect OAS3 expression and the associated inflammatory transcriptional program (50, 66). Glc and Gal culture conditions are commonly used to establish distinct metabolic contexts by altering the primary carbon source. Whereas glucose permits cells to rely predominantly on glycolysis, galactose culture promotes greater dependence on mitochondrial oxidative metabolism, thereby providing a widely used model for investigating the impact of cellular metabolism on macrophage function. In the present study, IFN-γ consistently induced OAS3 expression under both culture conditions, whereas stronger enrichment of multiple inflammation-related pathways was observed under Gal conditions than under Glc conditions. Previous studies have reported that IFN-γ promotes metabolic reprogramming in monocytes/macrophages and enhances mitochondrial oxidative phosphorylation (OXPHOS), thereby supporting inflammatory responses (30, 50). Together, these observations raise the possibility that differences in metabolic context may influence the overall IFN-γ-associated inflammatory transcriptional program, whereas OAS3 induction appears to be relatively preserved across the examined culture conditions. Because OAS3 was not experimentally manipulated in this dataset, the relationship between OAS3 and metabolic regulation remains untested and requires dedicated functional studies.

Given that OAS3 remained consistently induced under different metabolic conditions, we further integrated transcriptomic and epigenomic datasets to characterize molecular changes associated with its sustained responsiveness. Our analyses demonstrated that OAS3 expression was rapidly upregulated following IFN-γ stimulation; although its expression decreased after stimulus withdrawal, it remained elevated compared with the baseline condition, whereas treatment with the JAK inhibitor ruxolitinib further reduced OAS3 expression. Integrative analysis of ATAC-seq and CUT&Tag data revealed that IFN-γ stimulation was accompanied by increased chromatin accessibility and enhanced H3K4me1 enrichment at the OAS3 promoter region. These epigenetic features were partially maintained after IFN-γ withdrawal but were markedly attenuated following ruxolitinib treatment.

Previous studies have demonstrated that interferon signaling can promote STAT1-mediated chromatin opening through the JAK/STAT pathway and increase active enhancer-associated histone modifications, including H3K4me1, thereby facilitating promoter activation and sustaining the transcriptional activity of interferon-stimulated genes (58, 67–70). In addition, the JAK–STAT signaling pathway has been reported to directly regulate OAS3 promoter activity (71, 72). Together, these observations are consistent with an association between JAK/STAT signaling, altered chromatin features at the OAS3 locus, and sustained OAS3 expression. However, direct mechanistic studies are required to determine whether these chromatin changes mediate OAS3 induction.

To further explore the biological relevance of sustained OAS3 expression, we analyzed transcriptomic profiles of OAS3-ko cells. Because this public dataset included only one WT sample and two independent OAS3-ko clones, the observed pathway alterations should be interpreted as exploratory observations that generate hypotheses rather than as evidence of a direct functional role for OAS3.These exploratory observations suggest possible associations between OAS3 deficiency and neuroimmune transcriptional programs, which require validation in dedicated functional studies. Consistent with these observations, Previous studies have reported that elevated OAS3 expression is associated with neuroinflammation and cognitive impairment, and that its expression in peripheral monocytes parallels that in brain tissue, supporting its potential as a peripheral indicator of central immune activation (73, 74). OAS3 is also upregulated in the brains of patients with HIV-associated neurocognitive disorders and has recently been identified as a novel autoantigen in pSS (75, 76). Together, these findings support OAS3 as a candidate peripheral biomarker of immune activation. However, whether peripheral OAS3 reflects central neuroinflammatory activity or contributes to the shared immunopathology of pSS and AD remains to be determined.

This study has several limitations that should be acknowledged. First, the present work was primarily based on the integrative analysis of publicly available multi-omics datasets. Although the major findings were preliminarily validated by Western blot analysis and *in vitro* IFN-γ stimulation experiments, heterogeneity remained across datasets with respect to sample sources, disease stages, treatment status, and sequencing platforms. In addition, both candidate gene identification and the calculation of the IFN-γ score were based on a predefined IFN-γ-related gene set. Although these analyses addressed different biological questions and were performed using independent datasets, and therefore do not constitute circular analysis in the strict sense, the use of a shared gene set may have increased the correlation among the analytical results. Consequently, our findings require further validation in independent cohorts with larger sample sizes and more comprehensive clinical characterization.

Second, this study lacks direct causal and functional evidence supporting the shared immune features observed in pSS and AD. Our analyses primarily characterized shared IFN-γ-associated immune features from the perspective of peripheral immunity and did not directly evaluate key pathological events within the central nervous system, including neuroinflammation, microglial activation, or the accumulation of Aβ and tau pathology. Furthermore, the investigation of sustained OAS3 expression and its biological significance relied predominantly on publicly available multi-omics datasets and *in vitro* experiments. Because the public OAS3-knockout dataset included only one WT sample and two independent OAS3-knockout clones, the observed pathway alterations should be interpreted as exploratory observations that generate hypotheses rather than as evidence of a direct functional role for OAS3. Moreover, the relationship between OAS3 and cellular metabolism remains experimentally untested because OAS3 was not directly perturbed in the metabolic dataset analyzed in this study. Although JAK/STAT inhibition was associated with attenuation of the chromatin features accompanying OAS3 expression, whether these chromatin alterations directly regulate sustained OAS3 expression remains unknown.

Future studies incorporating OAS3 gain-of-function and loss-of-function models, disease-relevant animal models, integrative multi-omics analyses, and longitudinal clinical cohorts are needed to clarify the role of OAS3 in IFN-γ-associated immune regulation and the shared IFN-γ-associated immune features of pSS and AD. These studies will also be important for evaluating its biological significance and translational potential as a candidate biomarker.

In conclusion, by integrating multi-omics analyses with preliminary experimental validation, this study identified a shared IFN-γ-associated monocyte immune signature in pSS and AD and identified OAS3 as a candidate IFN-γ-responsive downstream molecule that exhibited sustained expression across the examined datasets and experimental conditions. Further analyses showed that sustained OAS3 expression was accompanied by altered chromatin accessibility and H3K4me1 enrichment at the OAS3 promoter, with these changes attenuated by ruxolitinib treatment, consistent with a possible involvement of JAK/STAT signaling. Collectively, these findings provide insights into the shared peripheral immune features of pSS and AD and support further investigation of OAS3 as a candidate molecule for mechanistic studies and as a potential peripheral marker of IFN-γ-associated immune activation.

## Data Availability

The original contributions presented in the study are included in the article/**Supplementary Material**. Further inquiries can be directed to the corresponding author/s.

## Supplementary material

Supplementary Figure Single-cell RNA-seq analysis.

Supplementary Table 1 Interferon-γ–Related Gene Set.

Supplementary Table 2 GEO Datasets.

Supplementary Table 3 Differentially Expressed Genes.

Supplementary Table 4 KEGG Analysis Results of 15 Co-upregulated DEGs.

Supplementary Table 5 Significant GSEA Pathways.

Supplementary Table 6 Peak Expression of OAS3.
