# Supplementary material for: Integrated multi-omics analysis reveals shared IFN-γ-associated monocyte signatures in primary Sjögren’s syndrome and Alzheimer’s disease
Source: Front Immunol. 2026 Sep 9;17:1923114. doi: 10.3389/fimmu.2026.1923114 (PMC13597436; doi:10.3389/fimmu.2026.1923114)
Supplement: Supplementary file 1 [file DataSheet1.pdf]

S1. Quality control and cell filtering of peripheral blood single-cell transcriptomic data in AD

After loading the peripheral blood single-cell RNA-seq dataset from AD patients (GSE181279), a systematic quality control (QC) analysis was performed. For each cell, the number of detected genes (nFeature_RNA), total transcript counts (nCount_RNA), proportion of mitochondrial genes (percent.mt), and proportion of ribosomal genes (percent.rb) were calculated. Prior to QC, the distributions of these metrics were relatively dispersed, with a subset of cells exhibiting abnormally high nCount_RNA and nFeature_RNA values, as well as a small fraction showing markedly elevated percent.mt, suggesting the presence of low-quality cells or potential doublets (Figure S1A).

Based on these distributions, the following filtering criteria were applied: nCount_RNA between 800 and 10,000, nFeature_RNA between 300 and 3,000, percent.mt ≤ 10%, and percent.rb ≤ 50%. After filtering, a total of 35,139 high-quality cells were retained, while 1,691 low-quality or aberrant cells were removed (approximately 4.6%). Post-filtering, all QC metrics showed markedly reduced dispersion, with extreme high values substantially diminished. In particular, cells with high percent.mt were effectively excluded (Figure S1B).

Further correlation analysis revealed a strong positive correlation between nCount_RNA and nFeature_RNA (r ≈ 0.91–0.92), indicating good concordance between sequencing depth and the number of detected genes. In contrast, nCount_RNA exhibited near-zero correlation with percent.mt (r ≈ −0.05 to −0.01), suggesting that mitochondrial gene proportion primarily reflects intrinsic cellular status rather than sequencing depth. A weak positive correlation was observed between nCount_RNA and percent.rb (r ≈ 0.17–0.21), consistent with the biological characteristics of ribosomal gene expression. Moreover, QC metric distributions were highly consistent across samples, with no evident systematic bias, indicating that the QC procedure effectively improved overall data reliability.

In summary, stringent quality control and filtering successfully removed low-quality cells and significantly enhanced the consistency and reliability of the single-cell dataset.

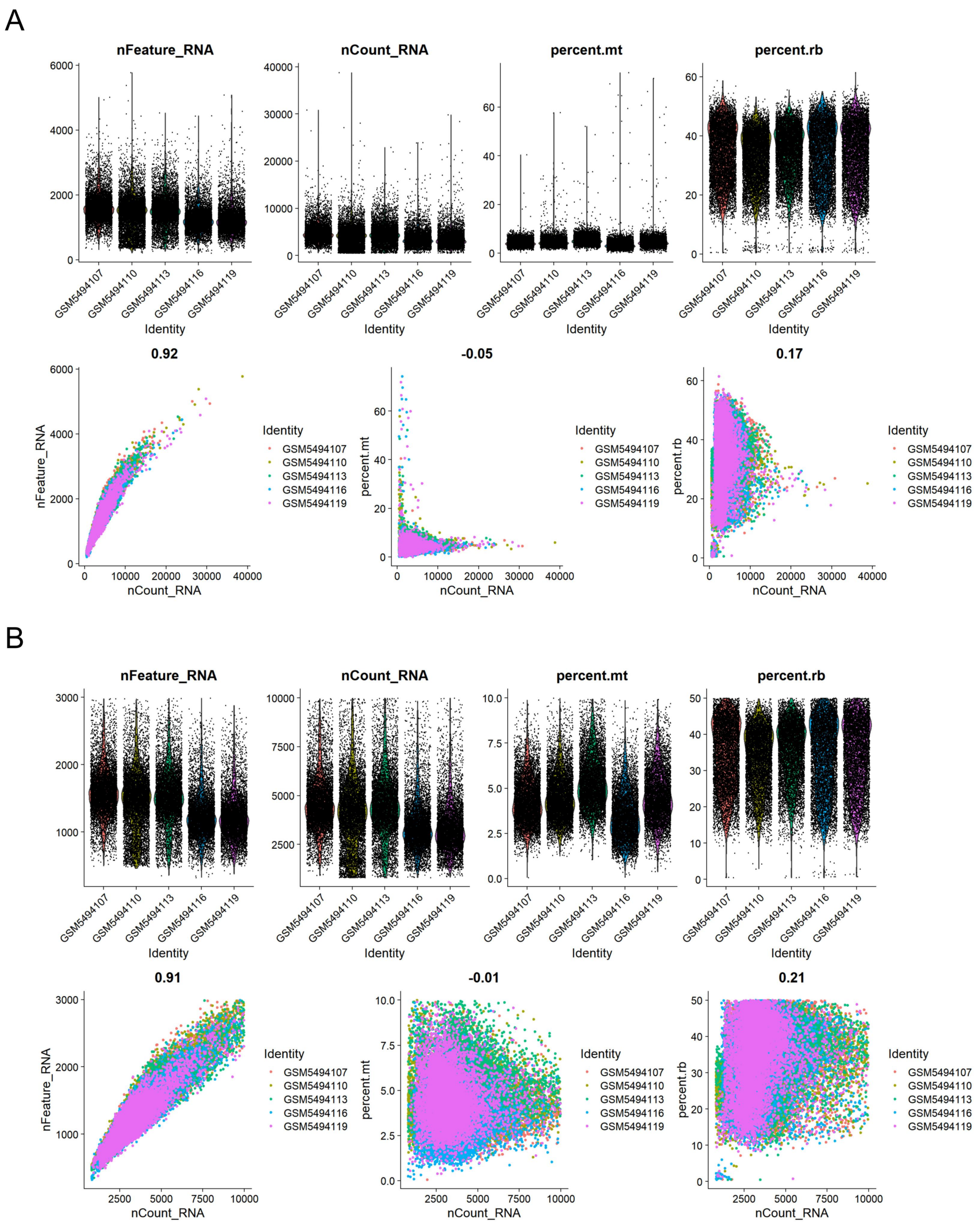

Figure S1 Quality control and filtering assessment of AD single-cell transcriptomic data.(A) Distributions of nFeature_RNA, nCount_RNA, percent.mt, and percent.rb across cells prior to QC.(B) Post-QC distributions showing reduced dispersion and effective removal of cells with extreme values, indicating improved data quality.

S2. Batch correction, dimensionality reduction, and clustering analysis of AD single-cell data

Following quality control, batch effects across samples were corrected using the Harmony algorithm, and highly variable genes were selected for downstream analyses. Principal component analysis (PCA) revealed that a certain degree of distributional heterogeneity remained among samples (Figure S2A).

Based on the Elbow plot, a pronounced decline was observed from PC1 to PC4, while the curve plateaued after PC12. Therefore, the first 12 principal components (PC1–PC12) were selected for subsequent dimensionality reduction and clustering (Figure S2B). Clustering stability under different resolution parameters was further evaluated using clustree analysis. The results indicated that when the resolution increased from 0.2 to 0.5, the lineage relationships among cell clusters remained relatively stable. In contrast, at resolutions ≥ 0.6, certain clusters exhibited evident splitting, suggesting potential over-clustering. Accordingly, a resolution of 0.5 was selected as the optimal parameter for clustering (Figure S2C).

Using these optimized parameters, UMAP visualization identified a total of 13 distinct cell clusters (Figure S2D). Furthermore, quality control metrics, including percent.mt, nFeature_RNA, and nCount_RNA, were evenly distributed across clusters, with no clusters driven by high mitochondrial content or extreme expression levels (Figure S2E), indicating that the clustering primarily reflects genuine biological variation.

In summary, through appropriate batch correction and parameter optimization, robust and reliable cell clustering was achieved.

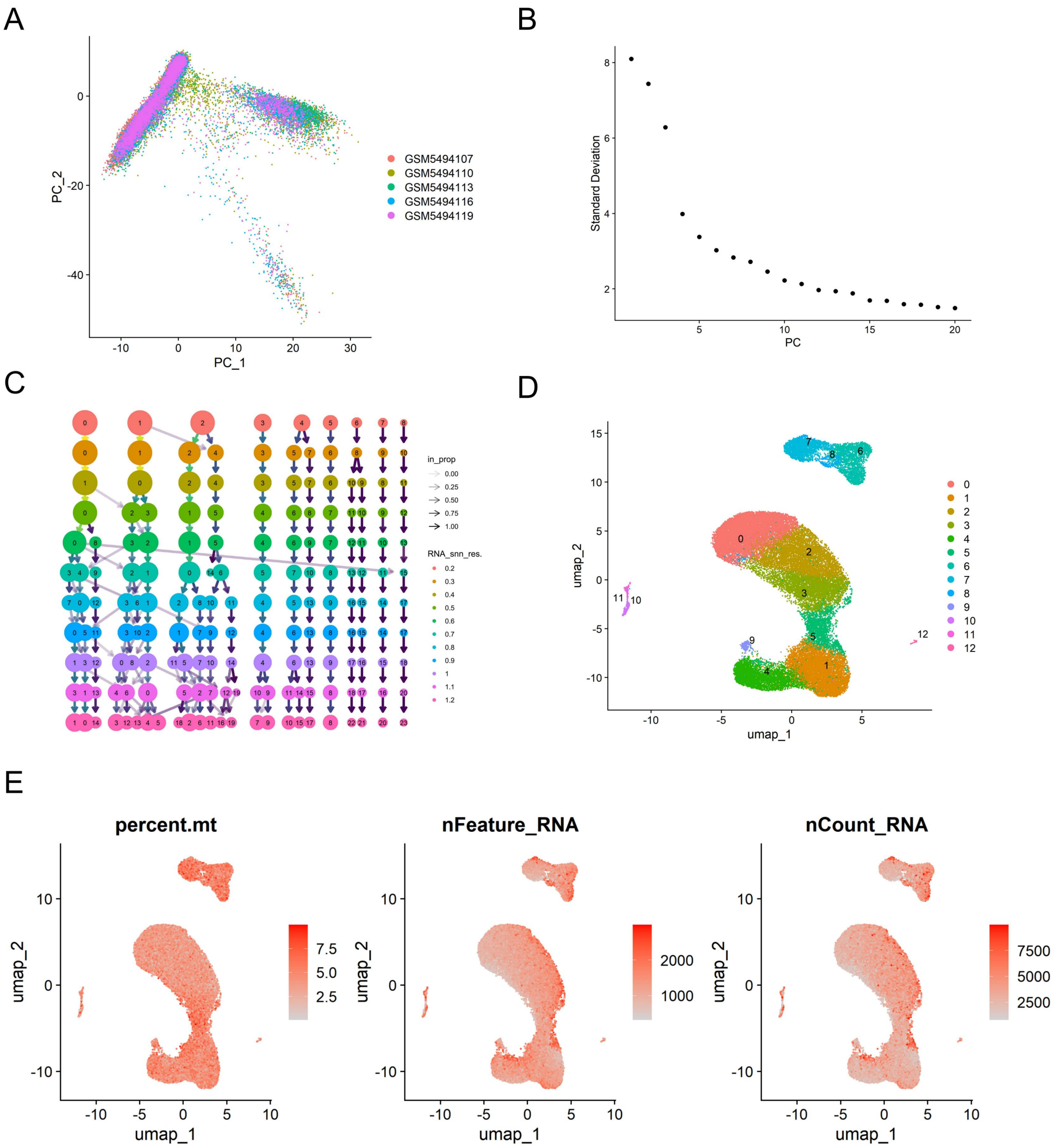

Figure S2.Dimensionality reduction, clustering, and QC metric distribution of AD single-cell transcriptomic data.(A) PCA plot showing the distribution of different samples in principal component space.(B) Elbow plot illustrating the inflection point used to determine the number of principal components.(C) Clustree plot displaying clustering stability and lineage relationships across different resolutions.(D) UMAP plot showing the clustering results of cells.(E) UMAP plots showing the spatial distribution of QC metrics (percent.mt, nFeature_RNA, and nCount_RNA).

S3. Annotation and subpopulation identification of peripheral immune cell subsets in AD

Based on dimensionality reduction and clustering of the single-cell transcriptomic data, a total of 13 cell clusters were identified. These clusters were manually annotated using canonical PBMC marker genes (PMID: 40112801).

Clusters 4 and 9 exhibited high expression of NK cell–associated genes, such as NKG7 and GNLY, and were therefore annotated as NK cells (Figure S3A). Among them, cluster 4 showed elevated expression of cytotoxicity-related genes, including GZMB and FCGR3A, and was classified as CD56^dim NK cells. In contrast, cluster 9 displayed higher expression of immunoregulatory genes, such as XCL1 and XCL2, and was classified as CD56^bright NK cells (Figure S3B).

Clusters 6, 7, and 8 were characterized by high expression of B cell marker genes, including MS4A1 and CD79A, and were annotated as B cells (Figure S3A). These were further subdivided into naïve B cells (TCL1A^{+}), memory B cells (CD27^{+}), and atypical memory B cells (S100A4^{+}, S100A10^{+}), reflecting substantial heterogeneity across different stages of B cell differentiation (Figure S3C).

Clusters 10 and 11 showed high expression of LYZ, S100A8, and S100A9, and were defined as monocytes (Figure S3A). These were further classified into CD14^{+} classical monocytes and CD16^{+} non-classical monocytes (Figure S3D).

Clusters 0, 1, 2, 3, and 5 exhibited high expression of T cell marker genes, including CD3D and CD3E, and were annotated as T cells (Figure S3A). Based on the expression of CD4 and CD8, these were divided into CD4^{+} T cells and CD8^{+} T cells. Further subdivision using functional markers (e.g., CCR7, CXCR5, IL23R, CX3CR1, and GZMK) revealed pronounced functional heterogeneity within the T cell compartment (Figure S3E, F).

In addition, cluster 12 displayed high expression of platelet-associated genes, such as PF4 and PPBP, and was annotated as platelets (Figure S3A).

Finally, cell populations were visualized using both UMAP and t-SNE dimensionality reduction methods (Figure S3G, H). The results demonstrated clear separation among major cell types in low-dimensional space, with well-defined cluster boundaries, further supporting the reliability of the cell type annotations.

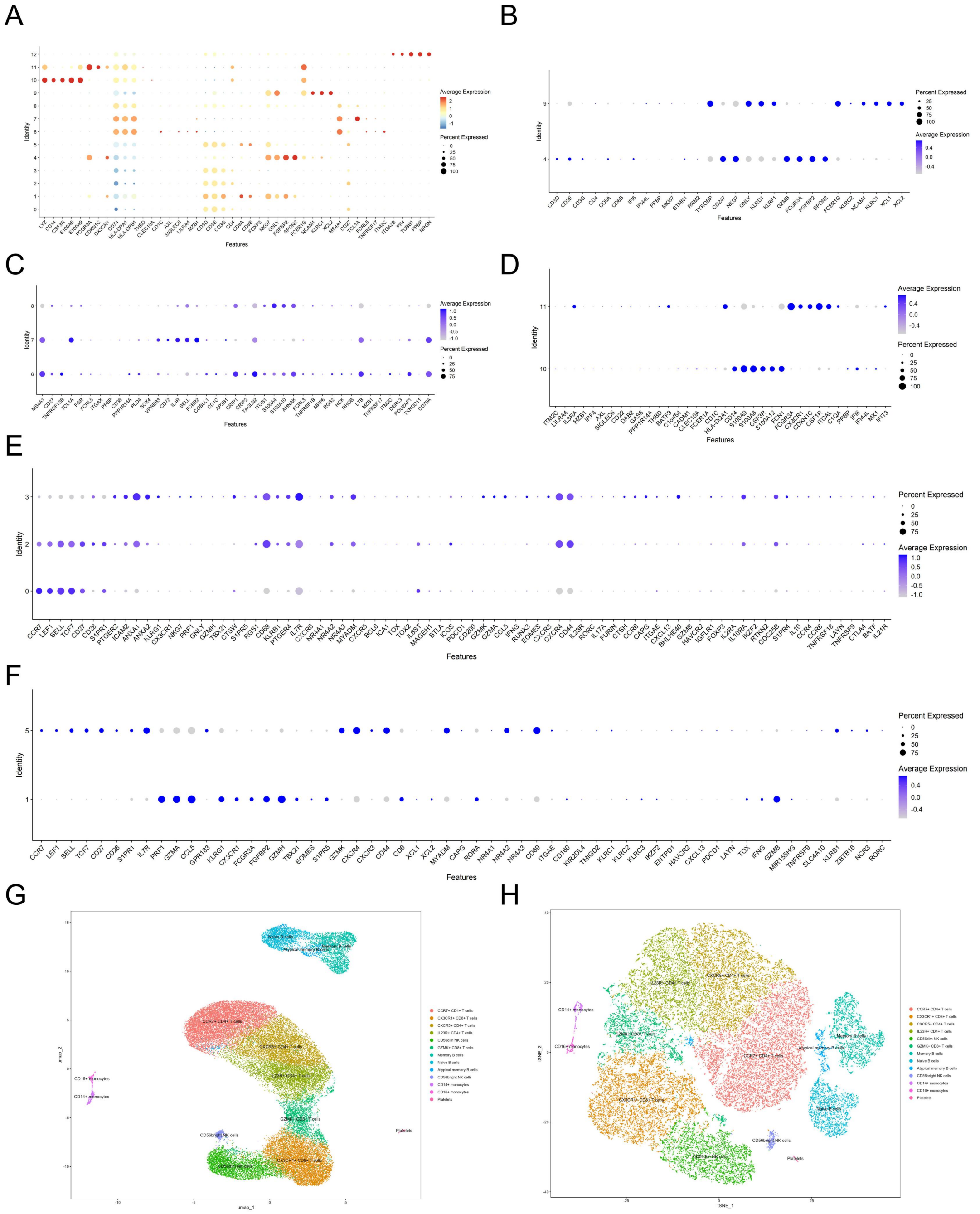

Figure S3.Composition of peripheral immune cells and characterization of major subpopulations in AD.(A) Bubble plot showing the expression of canonical marker genes across cell clusters for initial cell type annotation. Dot size represents the proportion of expressing cells, and color indicates average expression level.(B) Bubble plot of marker gene expression in NK cell subsets.(C) Bubble plot of marker gene expression in B cell subsets.(D) Bubble plot of marker gene expression in monocyte subsets.(E) Bubble plot of marker gene expression in $CD4^{+}$ T cell subsets.(F) Bubble plot of marker gene expression in $CD8^{+}$ T cell subsets.(G) UMAP plot showing the overall distribution and annotation of all cells.(H) t-SNE plot illustrating the spatial distribution of different cell types and relationships among clusters.

S4. IFNγ signaling activity and its cell type–specific distribution in AD

An IFNγ-related gene set was used to calculate module scores (AddModuleScore) for single-cell data, enabling a systematic characterization of IFN signaling from the perspectives of overall distribution, cell type–specific differences, and key gene expression.

In UMAP space, cells with high IFN scores exhibited a distinct clustered distribution, predominantly enriched in the monocyte compartment, while also present in subsets of T cells and NK cells (Figure S4A, B).

Significant differences in IFN scores were observed across cell types. $CD14^+$ and $CD16^+$ monocytes displayed the highest IFN activity, whereas NK cells (particularly CD56^bright NK cells) and a subset of $CD8^+$ T cells showed moderately elevated levels. In contrast, naïve $CD4^+$ T cells and platelets exhibited relatively low IFN scores (Figure S4C).

After stratifying cells into IFN-high and IFN-low groups, IFN-high cells were found to be predominantly enriched in monocytes, while showing a continuous gradient distribution across T and NK cell populations, suggesting a progressive activation pattern of IFN signaling within immune cells (Figure S4D).

Key IFN pathway genes, including IRF5, IRF7, and OAS3, were significantly upregulated in the IFN-high group and were primarily enriched in monocytes (Figure S4E–H). Among these, IRF7 displayed a broader expression pattern, reflecting overall activation of the IFN pathway, whereas IRF5 showed greater monocyte specificity, with particularly high expression in $CD16^+$ monocytes.

In summary, IFNγ signaling is prominently activated in monocytes in AD, suggesting a central role for these cells in IFN-mediated immune responses.

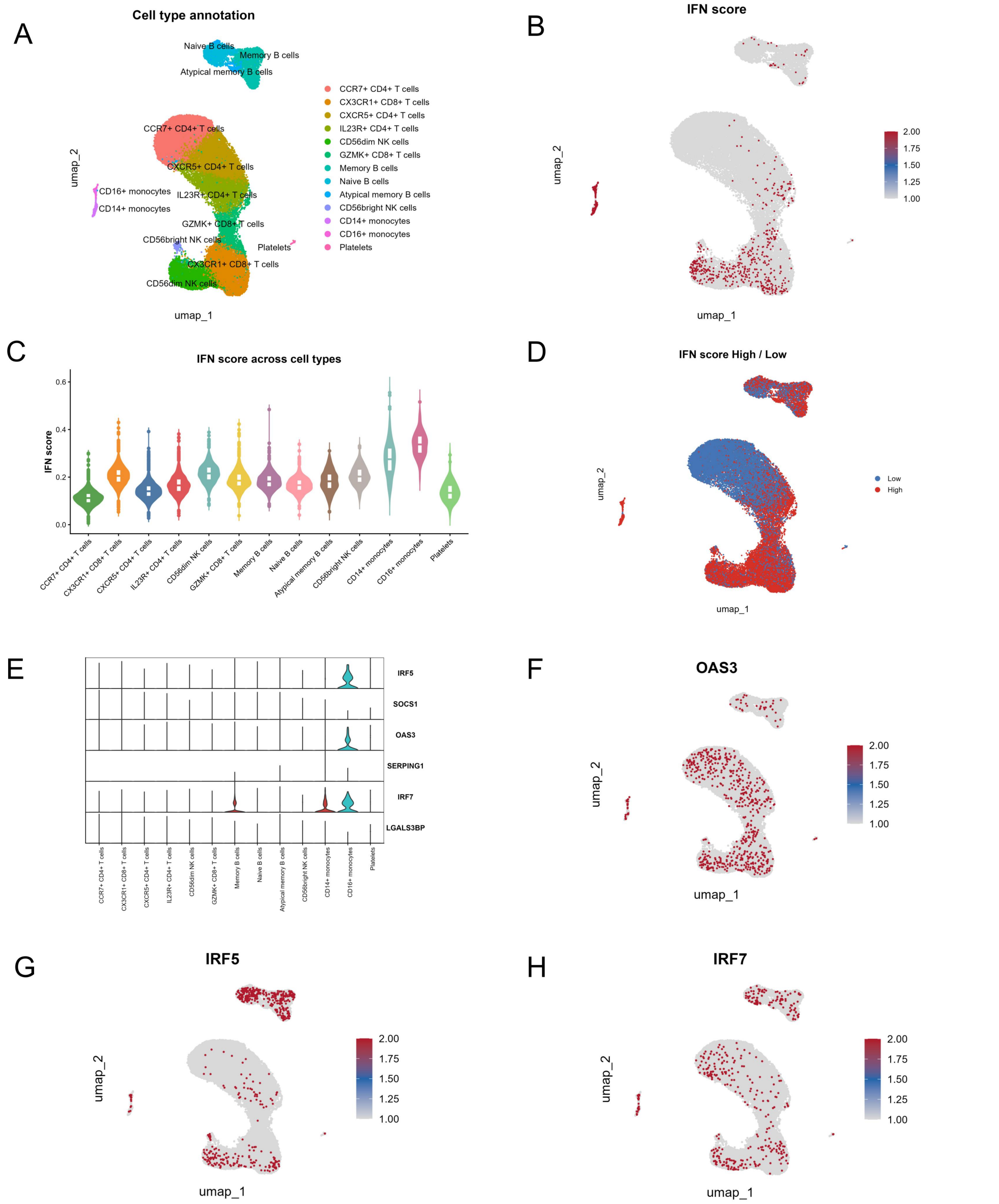

Figure S4. IFNγ signaling scores and expression patterns of key genes in AD single-cell data.(A) UMAP plot showing cell type annotations.(B) UMAP plot illustrating the distribution of IFNγ scores across cells, with color intensity indicating increasing IFNγ activity.(C) Violin plot showing the distribution of IFNγ scores across different cell types.(D) UMAP plot displaying IFNγ-high and IFNγ-low cells, with red indicating high expression and blue indicating low expression.(E) Violin plot showing the expression distribution of disease-related genes across cell types.(F) UMAP plot showing the expression pattern of OAS3.(G) UMAP plot showing the expression pattern of IRF5.(H) UMAP plot showing the expression pattern of IRF7.

S5. Quality control and cell filtering of pSS peripheral blood single-cell transcriptomic data

QC analysis was performed on the peripheral blood single-cell transcriptomic dataset from pSS (GSE157278). Prior to QC, nCount_RNA and nFeature_RNA exhibited a wide distribution range, and a subset of cells showed markedly elevated percent.mt values (exceeding 70%), indicating the presence of low-quality cells (Figure S5A). The following filtering thresholds were applied: nCount_RNA between 1,000 and 10,000, nFeature_RNA between 500 and 3,000, percent.mt ≤ 15%, and percent.rb ≤ 50%. After filtering, a total of 52,269 high-quality cells were retained, while 8,402 low-quality or outlier cells (13.8%) were removed (Figure S5B). Post-QC, the distributions of all metrics became markedly more constrained, and their correlations stabilized ($r \approx 0.88$, −0.2, and 0.23), indicating a substantial improvement in overall data quality (Figure S5B).

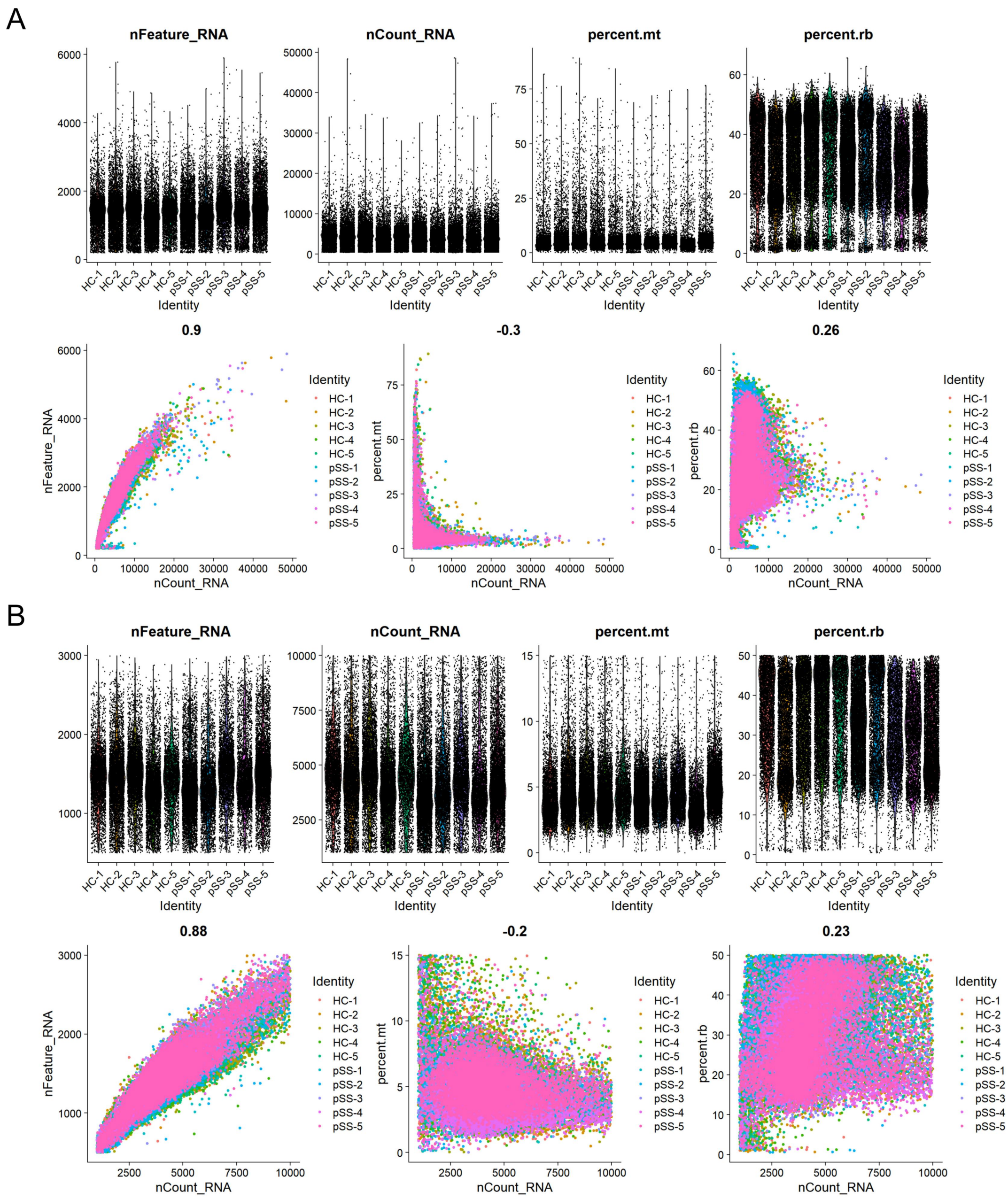

Figure S5.Quality control and filtering assessment of pSS single-cell transcriptomic data.(A) Distributions of nFeature_RNA, nCount_RNA, percent.mt, and percent.rb across cells prior to QC.(B) Post-QC distributions showing marked convergence of all metrics, with effective removal of outlier cells exhibiting extreme values, indicating improved data quality.

S6. Batch correction, dimensionality reduction, and clustering analysis of pSS single-cell data

Batch effects were corrected using Harmony, after which PCA results showed a marked reduction in inter-sample variability (Figure S6A). The elbow plot indicated that PC1–PC5 contributed most substantially to the variance; therefore, the top 10 principal components were selected for downstream analyses (Figure S6B). Clustree analysis demonstrated that clustering structures were stable within a resolution range of 0.3–0.4, whereas over-clustering was observed at resolutions ≥ 0.5. Accordingly, a resolution of 0.4 was chosen for subsequent analyses (Figure S6C). UMAP visualization identified a total of 10 distinct cell clusters (Figure S6D). Quality control metrics were evenly distributed across clusters, indicating that the clustering results were not driven by technical noise (Figure S6E).

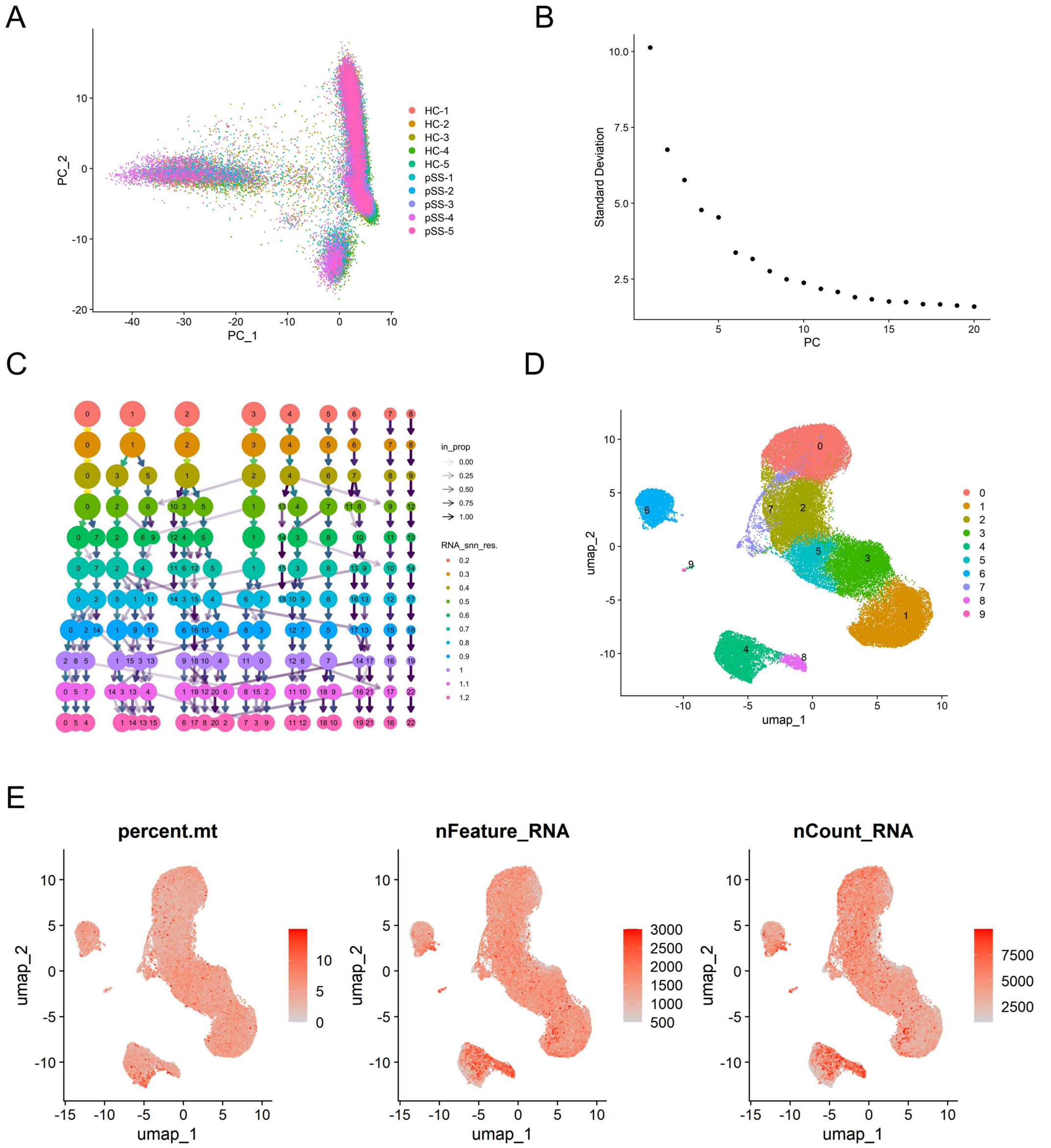

Figure S6.Dimensionality reduction, clustering, and QC metric distribution of pSS single-cell transcriptomic data.(A) PCA plot showing the distribution of samples in

principal component space.(B) Elbow plot used to determine the optimal number of principal components.(C) Clustree plot illustrating clustering stability and lineage relationships across different resolutions.(D) UMAP plot showing cell clustering results.(E) UMAP plots displaying the spatial distribution of QC metrics (percent.mt, nFeature_RNA, and nCount_RNA).

S7. Annotation and subpopulation identification of immune cell subsets in pSS peripheral blood

Based on dimensionality reduction and unsupervised clustering of the single-cell transcriptomic data, a total of 10 cell clusters were identified. Cell type annotation was performed manually using canonical PBMC marker genes reported in the literature (PMID: 40112801).

Cluster 1 exhibited high expression of NK cell–associated genes, including FCGR3A (CD16), NKG7, and GNLY, and was therefore annotated as CD16$^+$ NK cells (Figure S7A,B).

Within the B cell compartment, cluster 6 showed elevated expression of MS4A1 (CD20) and CD79A, and was classified as B cells with a memory-like phenotype (Figure S7A,C). In contrast, cluster 9 was characterized by high expression of plasma cell–specific genes, including MZB1, JCHAIN, and TNFRSF17, and was annotated as plasma cells (Figure S7A,C).

Clusters 4 and 8 both exhibited high expression of the ∂ ო ნ ocyte-associated gene LYZ. Based on differential expression of CD14 and FCGR3A, these clusters were annotated as CD14$^+$ classical monocytes and CD16$^+$ non-classical monocytes, respectively (Figure S7A,D).

Clusters 0, 2, 3, and 5 showed high expression of T cell marker genes CD3D and CD3E, and were collectively classified as T cells (Figure S7A). These were further subdivided into CD4$^+$ and CD8$^+$ T cell subsets according to CD4 and CD8 expression. Additional functional marker genes enabled finer classification into CCR7$^+$ CD4$^+$ T cells, GZMK$^+$ effector T cells, CX3CR1$^+$ CD8$^+$ T cells, and an SLC4A10-high CD8$^+$ T cell subset, highlighting the functional heterogeneity within the T cell compartment (Figure S7E,F).

Additionally, cluster 7 showed high expression of platelet-specific genes, including PPBP, PF4, and ITGA2B, and was annotated as platelets (Figure S7A).

Finally, UMAP and t-SNE were used to visualize the distribution of all cell populations (Figure S7G,H). The results demonstrated clear separation and well-defined boundaries among major cell types in low-dimensional space, further supporting the reliability of the cell annotations.

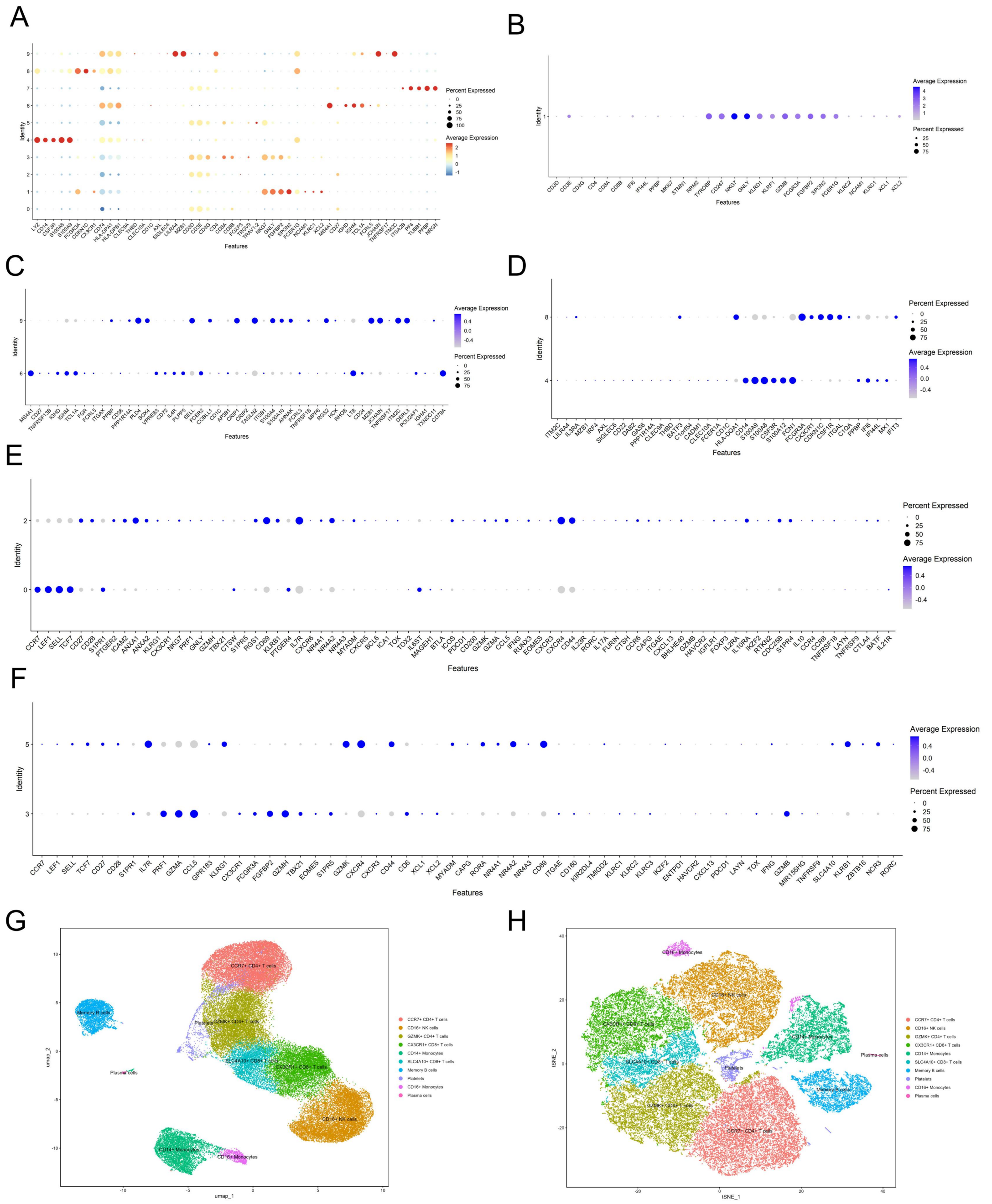

Figure S7.Composition of immune cells and characterization of major subpopulations in pSS peripheral blood.(A) Bubble plot showing the expression of canonical marker genes across clusters for initial cell type annotation. Dot size represents the proportion of expressing cells, and color indicates average expression level.(B) Bubble plot showing marker gene expression in NK cell subsets.(C) Bubble plot showing marker gene expression in B cell subsets.(D) Bubble plot showing marker gene expression in monocyte subsets.(E) Bubble plot showing marker gene expression in CD4^{+} T cell subsets.(F) Bubble plot showing marker gene expression in CD8^{+} T cell subsets.(G) UMAP plot showing the global distribution and annotation of all cells.(H) t-SNE plot showing the spatial distribution and relationships among different cell types.

S8. IFNγ signaling activity and its cell type–specific distribution in pSS

Following scoring of pSS single-cell transcriptomic data using an IFNγ-related gene set, monocyte populations—particularly $CD14^+$ and $CD16^+$ monocytes—exhibited the highest levels of interferon activity. In contrast, T cells and NK cells showed intermediate IFN scores, indicating a moderate interferon response with comparatively lower overall activity. B cells and platelets displayed generally low and narrowly distributed IFN signaling, suggesting limited pathway activation (Figure S8A–C).

Further stratification of cells into IFN-high and IFN-low groups based on IFN scores revealed that IFN-high cells were present across multiple immune cell types but were significantly enriched within the monocyte compartment, especially in the $CD16^+$

monocyte subset. This finding suggests that these cells may serve as the प्रमुख effector population in mediating IFN responses (Figure S8D).

At the gene expression level, OAS3, IRF5, and IRF7 exhibited expression patterns highly consistent with IFN scores, being predominantly enriched in IFN-high regions and particularly elevated in monocytes (Figure S8E–H). Among these, IRF7 showed relatively broad expression across cell types, with intensity dependent on IFN status, whereas IRF5 displayed a stronger cell type bias, being preferentially enriched in monocytes—especially $CD16^+$ monocytes (Figure S8F–H).

Collectively, these results indicate that IFNγ-related signaling is predominantly activated in monocytes in pSS, with the most pronounced activity observed in $CD14^+$ and $CD16^+$ subsets, consistent with findings from AD analyses.

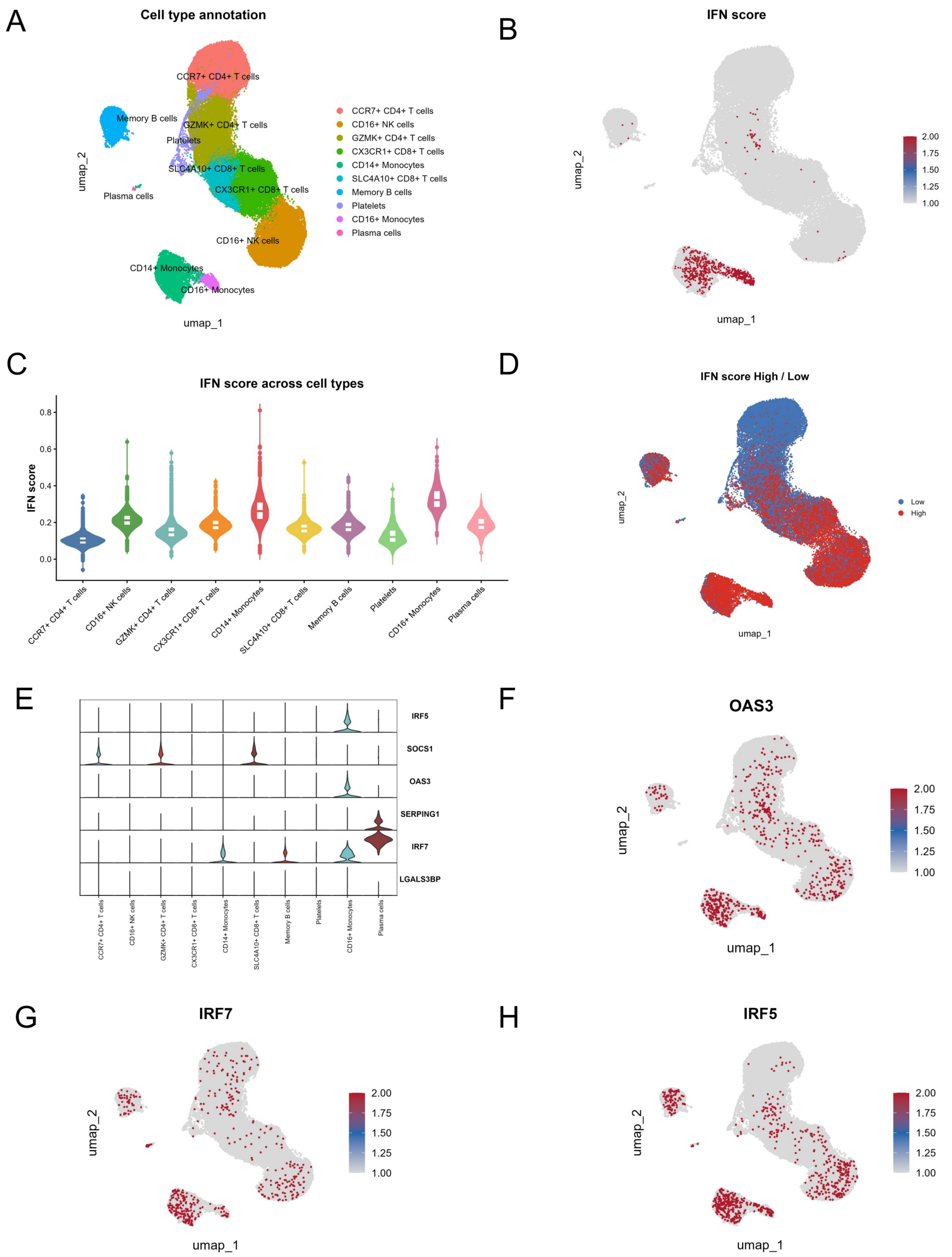

Figure S8.IFNγ signaling scores and key gene expression patterns in pSS single-cell data.(A) UMAP plot showing cell type annotations.(B) UMAP plot displaying the distribution of IFNγ scores across cells, with color gradients from low to high.(C) Violin plot illustrating the distribution of IFNγ scores across different cell types.(D) UMAP plot showing the distribution of IFNγ-high (red) and IFNγ-low (blue) cells.(E) Violin plot showing the expression distribution of comorbidity-associated genes across cell types.(F) UMAP plot showing the expression pattern of OAS3.(G) UMAP plot showing the expression pattern of IRF7.(H) UMAP plot showing the expression pattern of IRF5.

S9. Dynamic changes in chromatin accessibility and H3K4me1 modification at the OAS3 locus in Subject 2

To further validate the epigenetic changes observed at the OAS3 locus in Subject 1, ATAC-seq (GSE294915) and H3K4me1 CUT&Tag (GSE294916) data from an independent healthy donor (Subject 2) were analyzed separately. Because peak calling and data processing were performed independently for each donor, this analysis was intended solely for descriptive comparison and no statistical inference was performed across donors.

In the ATAC-seq dataset, a single major chromatin accessibility peak was identified within the OAS3 locus, corresponding to the promoter region (Figure S9A). Dynamic analysis showed that chromatin accessibility at this promoter-associated peak increased after 8 h of IFN-γ stimulation and remained elevated following cytokine withdrawal, whereas ruxolitinib treatment was associated with a reduction in chromatin accessibility (Figure S9B). This dynamic pattern was consistent with the IFN-γ-induced increase in chromatin accessibility observed in Subject 1.

Analysis of the H3K4me1 CUT&Tag dataset identified multiple H3K4me1-enriched peaks across the OAS3 locus, including peaks located within the promoter and adjacent regulatory regions (Figure S9C). Consistent with the ATAC-seq results, several H3K4me1 peaks exhibited increased enrichment following IFN-γ stimulation, which remained relatively elevated after cytokine withdrawal, whereas this trend was attenuated following ruxolitinib treatment (Figure S9D).

Taken together, Subject 2 exhibited dynamic changes in chromatin accessibility and H3K4me1 enrichment at the OAS3 promoter that were consistent with those observed in Subject 1, providing additional descriptive support for the association between IFN-γ stimulation and epigenetic remodeling of the OAS3 regulatory region during transcriptional activation.

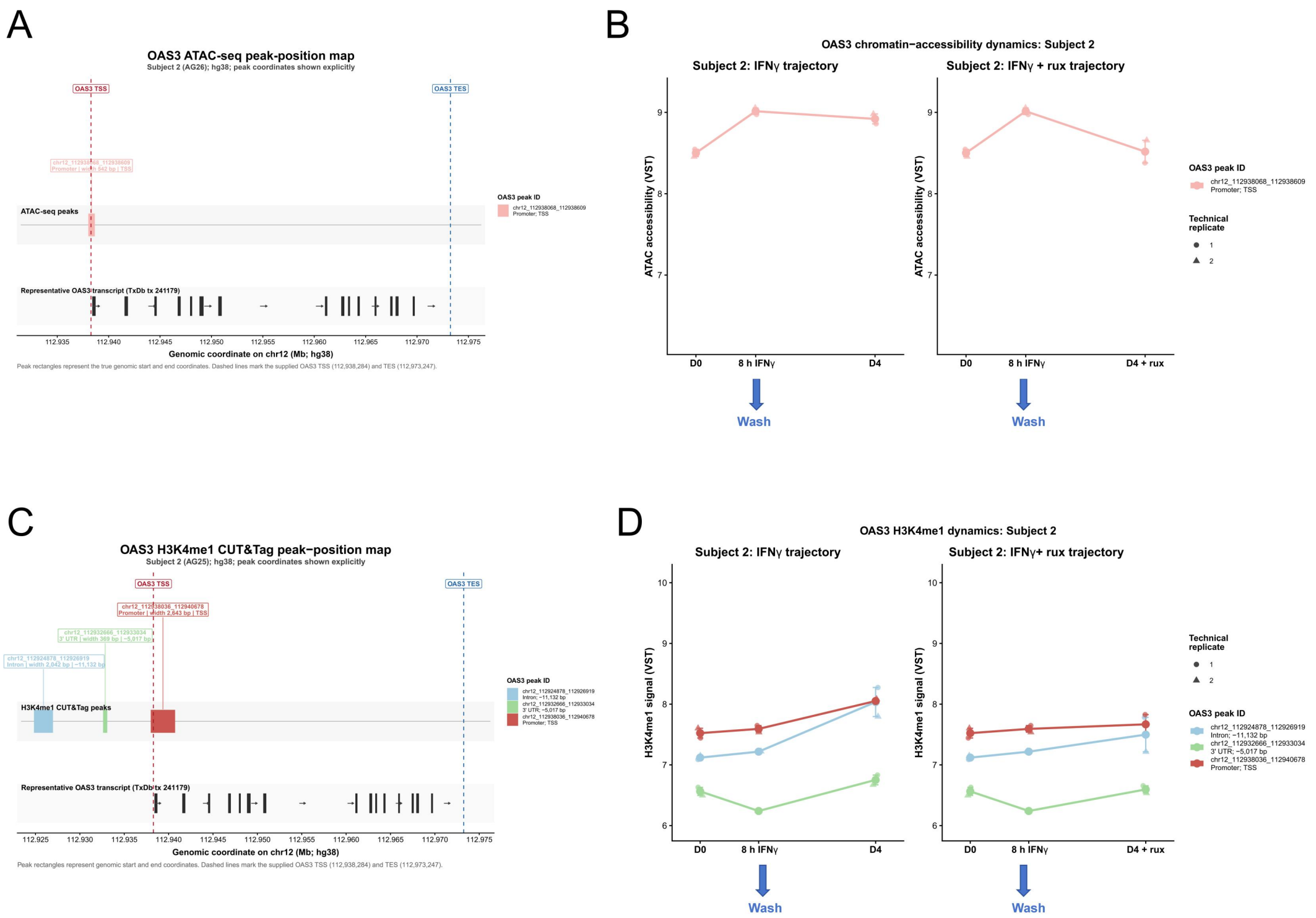

Figure S9.Dynamic changes in chromatin accessibility and H3K4me1 modification at the OAS3 locus in Subject 2.(A) Genomic distribution of ATAC-seq peaks at the OAS3 locus (hg38, chr12) in Subject 2. Colored boxes indicate the genomic locations and annotation categories of ATAC-seq peaks, including promoter (red), 3′ untranslated region (3′ UTR, green), intron (blue), and other regions (gray-purple). The representative OAS3 transcript and exon structure are shown below. Arrows indicate the direction of transcription, and dashed lines denote the transcription start site (TSS) and transcription end site (TES).(B) Dynamic changes in chromatin accessibility of the OAS3 promoter-associated ATAC-seq peak in Subject 2 under different treatment conditions. The left panel shows the IFN-γ stimulation and washout trajectory (IFNγ trajectory), whereas the right panel shows the IFN-γ stimulation followed by ruxolitinib treatment (IFNγ + rux trajectory). Data points represent the mean variance-stabilized transformation (VST) values of two technical replicates, and error bars indicate variability between technical replicates.(C) Genomic distribution of H3K4me1 CUT&Tag peaks at the OAS3 locus in Subject 2 . Colored boxes indicate the genomic locations and annotation categories of H3K4me1-enriched peaks, including promoter (red), 3′ untranslated region (3′ UTR, green), intron (blue), and other regions (gray-purple). The representative OAS3 transcript and exon structure are shown below. Arrows indicate the direction of transcription, and dashed lines denote the TSS and TES.(D) Dynamic changes in H3K4me1 enrichment at the OAS3 locus in Subject 2 (AG25) under different treatment conditions. The left panel shows the IFN-γ stimulation and washout trajectory (IFNγ trajectory), whereas the right panel shows the IFN-γ stimulation followed by ruxolitinib treatment (IFNγ + rux trajectory). Different colors represent

distinct H3K4me1 peaks. Data points represent the mean VST values of two technical replicates, and error bars indicate variability between technical replicates.
